# Comparative Effectiveness of Abatacept Versus Adalimumab in Shared Epitope Positive and Negative Patients With Rheumatoid Arthritis

**DOI:** 10.1002/art.43298

**Published:** 2025-09-23

**Authors:** Chuan Fu Yap, Nisha Nair, Seema D. Sharma, John Bowes, Amirah Binti Mohammad Ariff, Ann W. Morgan, John D. Isaacs, Anthony G. Wilson, Kimme L. Hyrich, Suzan Verstappen, James Bluett, Andrew P. Morris, Anne Barton, Darren Plant, Sebastien Viatte

**Affiliations:** ^1^ Centre for Genetics and Genomics Versus Arthritis, The University of Manchester Manchester United Kingdom; ^2^ Centre for Genetics and Genomics Versus Arthritis, The University of Manchester and NIHR Manchester Biomedical Research Centre, Manchester University NHS Foundation Trust, Manchester Academic Health Science Centre Manchester United Kingdom; ^3^ School of Medicine, University of Leeds and NIHR Leeds Biomedical Research Centre, Leeds Teaching Hospitals NHS Trust Leeds United Kingdom; ^4^ Translational and Clinical Research Institute, Newcastle University and Musculoskeletal Unit and NIHR Newcastle Biomedical Research Centre, Newcastle‐upon‐Tyne Hospitals NHS Foundation Trust Newcastle‐upon‐Tyne United Kingdom; ^5^ School of Medicine and Medical Science, Conway Institute, University College Dublin Dublin Ireland; ^6^ Centre for Epidemiology Versus Arthritis, Centre for Musculoskeletal Research, Division of Musculoskeletal and Dermatological Sciences, The University of Manchester and NIHR Manchester Biomedical Research Centre, Manchester University NHS Foundation Trust, Manchester Academic Health Science Centre Manchester United Kingdom; ^7^ Centre for Genetics and Genomics Versus Arthritis and Lydia Becker Institute of Immunology and Inflammation, Faculty of Biology, Medicine and Health, The University of Manchester and NIHR Manchester Biomedical Research Centre, Manchester University NHS Foundation Trust, Manchester Academic Health Science Centre Manchester United Kingdom

## Abstract

**Objective:**

The effect of the shared epitope (SE) and valine at position 11 (Val11) of HLA–DRB1 on the activation of CD4^+^ T cells is expected to be diminished by abatacept, a costimulation blocker. However, published evidence on the value of genetic stratification for abatacept treatment is conflicting. We aimed to compare the difference in effectiveness of abatacept and adalimumab in patients carrying the SE (or Val11).

**Methods:**

The Biologics in Rheumatoid Arthritis Genetics and Genomics Study Syndicate is a nationwide observational cohort study recruiting patients from 53 centers across the United Kingdom before the initiation of biologic treatment and following them up prospectively for 12 months. Three hundred forty‐two patients starting either abatacept or adalimumab were eligible for this analysis. Serum drug levels for abatacept, adalimumab, and methotrexate were determined at multiple time points. Multivariate modeling integrating demographic, clinical, and pharmacological variables was used to test for associations between the number of copies of the SE or Val11 and response to treatment (EULAR response; Disease Activity Score in 28 joints [DAS28] remission; change in DAS28). Differential effectiveness between drugs and genetic markers was assessed by the significance of their interaction term.

**Results:**

There was no difference in the efficacy of abatacept versus adalimumab. We found weak evidence for an independent association of genetic markers with response to treatment (Val11 with EULAR response: *P* = 0.02), but there was no significant difference in this effect between drugs.

**Conclusion:**

We found no evidence that HLA typing is clinically useful to support prescription decisions for these two drugs.

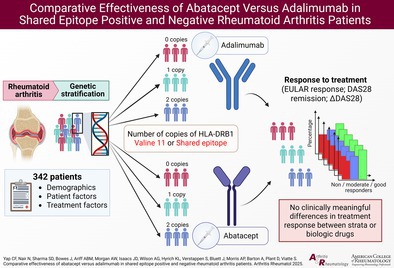

## INTRODUCTION

Biologic drugs licensed for the treatment of rheumatoid arthritis (RA) act on very different pathways, with no drug being universally effective.^1^ In a subset of patients, autoreactive CD8^+^ and CD4^+^ T cells^2^, ^3^, ^4^ arise through the presentation of citrullinated self‐antigens on major histocompatibility complex (MHC) class I and MHC class II molecules,^5^, ^6^ some of which are encoded by so called HLA–DRB1 shared epitope (SE) alleles.^7^ In addition, T cell activation requires costimulation provided by the interaction between CD80/CD86 on antigen‐presenting cells and CD28 on T cells, a process blocked by abatacept. A large number of reports focusing on anti–citrullinated protein/peptide antibody (ACPA)–positive RA, in which the SE is present in up to 80% of patients,^6^, ^8^, ^9^ have shown a better responsiveness to abatacept versus tumor necrosis factor inhibitors (TNFi) in ACPA‐positive RA^10^, ^11^ or in patients with higher ACPA titers.^12^

The assessment of a direct effect of genetics on the differential effectiveness of abatacept, however, has led to conflicting reports. In four independent studies,^13^, ^14^, ^15^, ^16^ the carriage of the SE or individual HLA–DRB1 susceptibility alleles was significantly associated with an improved response to abatacept but not to TNFi or JAK/STAT inhibitors (TOF‐ABT Study^13^), and this effect was independent of ACPA status. In addition, a clear dose–response effect for each copy of the SE was observed in the abatacept group but not in the tofacitinib group.^13^ By contrast, three recently published studies^17^, ^18^, ^19^ and the phase 3 AMPLIFIED trial (an abatacept versus adalimumab head‐to‐head comparison)^20^ failed to show a statistically significant improvement in abatacept response in patients carrying SE alleles. Interestingly, a recent study by Cha et al in Korean patients has shown that valine at position 11 (Val11) of HLA–DRB1, outside the SE, was more strongly associated with response to abatacept than the SE itself.^17^

It is currently not clear if the search for an abatacept endotype should be based on the SE or on other susceptibility alleles,^21^ combined or not with serologic status. Moreover, adherence and drug levels, which could be important confounders in studies of treatment response,^22^, ^23^, ^24^, ^25^ have not been considered in studies of differential drug effectiveness in patients carrying SE alleles.

Here, we aim to compare the difference in effectiveness of abatacept versus adalimumab for each copy of the SE or Val11 in patients from the Biologics in Rheumatoid Arthritis Genetics and Genomics Study Syndicate (BRAGGSS) cohort by adjusting for potential confounders and known clinical predictors of response, including drug levels for methotrexate (MTX), abatacept, and adalimumab.

## METHODS

### Patients and cohort

BRAGGSS recruited patients with a clinical diagnosis of RA from 53 centers across England from November 2008 to December 2019. All patients provided written informed consent (ethical approval COREC 04/Q1403/37). Patients with active disease (Disease Activity Score in 28 joints [DAS28] > 5.1) who failed to respond to conventional synthetic disease‐modifying antirheumatic drugs were recruited and assessed at baseline, before the initiation of a first‐line biologic treatment or a new biologic treatment (second line or higher). Participants were observed prospectively with assessments at 3, 6, and 12 months. Baseline variables were age, sex, disease duration, body mass index (BMI), patient‐reported comorbidities, Health Assessment Questionnaire score, and DAS28 components (C‐reactive protein [CRP] or erythrocyte sedimentation rate [ESR], swollen joint count, tender joint count, and patient or physician global assessment on a visual analog scale [VAS; 0–100]). Follow‐up clinical assessments included the DAS28 and its subcomponents. Blood samples were collected to allow measurement of drug levels. Inclusion criteria for this study included the initiation of a biologic treatment with either abatacept or adalimumab and the availability of DNA and treatment response data for at least one follow‐up time point within the first six months of treatment. All patients receiving abatacept (as a first‐line biologic treatment, second line or higher order) in the BRAGGSS database^26^ were selected for this study, and an equivalent number of patients receiving adalimumab fulfilling the same inclusion criteria were also included as a comparison group.

### Treatment response

Treatment response was determined using one of the three following measures: (1) the EULAR criteria, as an ordinal variable^27^; (2) DAS28 remission, as a binary variable (DAS28 < 2.6); and (3) ΔDAS28, as a continuous variable (DAS28 at follow‐up minus DAS28 at baseline). Response was computed using the four‐component DAS28‐CRP (or the three‐component DAS28 if the VAS was missing) at six months after treatment initiation or the DAS28‐ESR if the DAS28‐CRP was unavailable. If both the DAS28‐CRP and the DAS28‐ESR were missing at six months, then the three‐month DAS28 was used. This approach was used because EULAR responses computed using scores at three and six months were strongly correlated in this dataset (Pearson r = 0.83, *P* = 3.5 × 10^−269^).

### Genotyping, quality control, imputation, and definition of the SE

Genotype data were generated according to the manufacturer's protocol using the Illumina Infinium CoreExome‐24 v1.4 array on the Illumina iScan. Quality control of genotype data was performed using standard thresholds: exclusion of single‐nucleotide polymorphisms (SNPs) with a low call rate (>2% missing), with a minor allele frequency <1% or out of Hardy–Weinberg equilibrium (*P* < 1 × 10^−4^); exclusion of samples with >2% missing SNPs, with a heterozygosity rate greater than 3 SDs from the sample mean; or exclusion of duplicated samples or related individuals, as determined by identity by descent, or individuals with a genetically determined sex differing from the recorded sex. Finally, to avoid spurious associations due to population stratification, the genetic ancestry of each individual was determined using the HapMap 3 reference panel, and only individuals of European ancestry were retained for downstream analyses. Phasing, genotype and HLA imputation, and the definition of HLA–DRB1 alleles considered as SE alleles are presented in the Supplementary Methods.

### Drug levels

We have previously demonstrated the value of nontrough levels of biologic drugs in predicting response to treatment.^28^ Nontrough drug levels for abatacept or adalimumab were measured at three and six months using sandwich enzyme‐linked immunosorbent assay kits (Sanquin Diagnostic Services). For data analysis purposes, adalimumab and abatacept drug levels below the detection limit of 0.01 μg/mL were set to 0 μg/mL. Abatacept drug levels above 160 μg/mL were changed to 160 μg/mL because these levels are above the dynamic range of the test. The availability and distribution of biologic drug levels in this study, together with their median levels at each time point, is presented in Supplementary Table 1 and Supplementary Figure 1. Adalimumab and abatacept drug levels were correlated between the three‐ and six‐month measurements but had a nonnormal distribution with a plateau. Based on this observation and the pharmacokinetics of each drug (including published evidence on the association of nontrough drug levels with response),^28^, ^29^, ^30^ we decided to (1) dichotomize drug levels using a 5‐μg/mL cutoff for adalimumab and a 10‐μg/mL cutoff for abatacept, as levels below these cutoff values are associated with nonresponse, and (2) use the six‐month measurement for data analysis and, only if missing, the three‐month measurement. Therefore, we referred to the “presence/absence of therapeutic drug levels” when the dichotomized variable was used.

Because both MTX use^26^ and inadequate adherence to MTX treatment^23^ are known to be associated with response to biologic drugs, we determined serum MTX levels in patients eligible for this study. We used the World Health Organization definition of adherence: “the extent to which a person's behaviour – taking medication, following a diet, and/or executing lifestyle changes, corresponds with agreed recommendations from a health care provider.”^31^, ^32^ When serum was available, MTX nontrough levels were determined at baseline, three months, and six months using a sensitive liquid chromatography tandem mass spectrometry method as previously described.^33^, ^34^ Supplementary Table 2 and Supplementary Figure 1 describe in detail the concordance between measured levels and the clinical variable “concomitant prescription of MTX,” and the method to impute one from the other is outlined in the Supplementary Methods. Throughout this article, “MTX use” refers to the concomitant prescription of MTX, which, when missing from the databank, was imputed based on measured MTX levels; it is a categorical variable (0 = no use; 1 = use of MTX). Measured MTX levels were also used to relabel MTX use by biochemically nonadherent patients, whereby nonadherence was defined for this analysis as undetectable levels of MTX at all measured time points for a patient prescribed MTX according to the clinician questionnaires.

### Statistical framework for association testing and selection of covariates

Analysis was conducted in accordance with Strengthening the Reporting of Observational Studies in Epidemiology and EULAR points to consider for comparative effectiveness research.^35^, ^36^ To test for differences between adalimumab‐treated and abatacept‐treated patients in Table 1, we used a *t*‐test for continuous values, and we modeled categorical values with the chi‐square test and nonnormally distributed variables with the Kruskal–Wallis test. Multiple imputation by chained equations^37^ was used to impute missing BMI data with 100 iterations of imputation, and effect sizes (coefficients) were pooled using Rubin's rule.

**Table 1:** Cohort characteristics of BRAGGSS patients included in this study*

	Missing, n (%)	Overall	Abatacept	Adalimumab	*P* value
n	–	342	136	206	
Female sex, n (%)	0	265 (77.5)	106 (77.9)	159 (77.2)	0.98
Age, mean (SD), y	0	58.5 (11.8)	60.6 (12.3)	57.1 (11.3)	0.008
ACPA positive, n (%)	194 (56.7)	99 (66.9)	12 (60)	87 (68)	0.65
Biologic naïve, n (%)	0	180 (52.6)	38 (27.9)	142 (68.9)	<0.001
Disease duration, median (Q1, Q3), y	7 (2)	9.6 (3.1, 17.6)	12.6 (5.0, 21.4)	7.0 (2.5, 15.6)	<0.001
Baseline DAS28, median (Q1, Q3)	13 (3.8)	5.5 (5.0^a^, 6.1)	5.5 (5.0, 6.1)	5.5 (5.1, 6.1)	0.63
MTX usage, n (%)	0	237 (69.3)	87 (64.0)	150 (72.8)	0.11
Concurrent DMARDs usage, n (%)	0	276 (80.7)	107 (78.7)	169 (82.0)	0.53
Comorbidity status, n (%)	0	89 (26.0)	50 (36.8)	39 (18.9)	<0.001
BMI, median (Q1, Q3)	57 (16.7)	28.1 (24.3, 32.8)	28.3 (25.0, 32.8)	28.0 (24.1, 32.8)	0.29
Shared epitope status, n (%)					0.11
0	0	69 (20.2)	24 (17.6)	45 (21.8)	
1	–	181 (52.9)	67 (49.3)	114 (55.3)	
2	–	92 (26.9)	45 (33.1)	47 (22.8)	
EULAR response, n (%)					
None	37 (10.8)	75 (24.6)	38 (34.5)	37 (19.0)	0.001
Moderate	–	109 (35.7)	42 (38.2)	67 (34.4)	
Good	–	121 (39.7)	30 (27.3)	91 (46.7)	
Remission status, n (%)	0	84 (24.6)	22 (16.2)	62 (30.1)	0.005
Change in DAS28, median (Q1, Q3)	49 (14.3)	2.1 (0.9, 3.0)	1.5 (0.7, 2.6)	2.2 (1.3, 3.2)	0.001

* Statistical tests are described in the Methods. ACPA, anti–citrullinated protein/peptide antibody; BMI, body mass index; BRAGGSS, Biologics in Rheumatoid Arthritis Genetics and Genomics Study Syndicate; DAS28, Disease Activity Score in 28 joints; DMARD, disease‐modifying antirheumatic drug; MTX, methotrexate; n, number of observations; Q1, Q3, first and third quartile.

^a^ Although BRAGGSS recruitment criteria include a minimum DAS28 of 5.1, 90 patients in this study had a baseline DAS28 <5.1.

**Figure 1:**
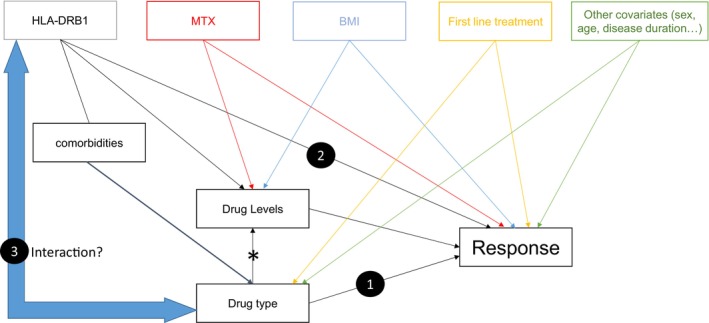
Known and expected associations with response to treatment, including direct and indirect effects measured in this study. This figure depicts our research hypothesis (testing of relationships 1 and 2 and their interaction [3]) alongside relevant covariates. HLA–DRB1 represents two different independent variables^38^ (number of copies [either 0, 1, or 2 carried by each participant] of the SE or of Val11). ACPA may be a path variable for relationship 2 and was therefore not included,^43^ but a separate subanalysis of ACPA‐positive patients was conducted (Supplementary Table 9). Association of HLA–DRB1 (relationship 2) has been observed for TNFi.^38^ MTX use and sex are known to be associated with response.^26^ Association between BMI and drug levels and response has previously been reported.^28^ *Serum drug levels for different medications are not on the same scale. ACPA, anti–citrullinated protein/peptide antibody; BMI, body mass index; MTX, methotrexate; SE, shared epitope; TNFi, tumor necrosis factor inhibitor; Val11, valine at position 11.

To test if abatacept was significantly more effective than adalimumab for each copy of the SE (or Val11), a multivariate model was used. We assessed the association of biologic drug type, number of copies of the SE (or Val11), and the interaction between the SE (or Val11) and biologic drug type (relationships 1, 2, and 3, respectively; Figure 1) with each of the three measures of treatment response. In this setting, the interaction term represents the differential effectiveness of adalimumab and abatacept in different genetic strata.

Relevant covariates were selected based on the current literature^26^, ^28^, ^38^, ^39^ and are depicted in Figure 1 (results from univariate associations with response to treatment are presented in Supplementary Tables 3–5). Covariates displayed a low level of correlation (Supplementary Figure 2). Therefore, we adjusted for sex, age, disease duration, BMI, MTX use, presence/absence of therapeutic drug levels, and whether patients were biologic naïve. In the United Kingdom, British Society of Rheumatology guidelines suggest using abatacept as a first‐line biologic therapy in patients at high risk of infection.^40^ Reflecting this, patients prescribed abatacept tend to be older and have more comorbidities in comparison to patients prescribed adalimumab. In addition, the presence of comorbidities is associated with both the exposure (genetic markers) and the outcome (response to treatment) in the dataset. Therefore, we also adjusted the multivariate model for the presence of comorbidities.

The variable “biologic naïve” is defined as a categorical variable, used to distinguish between patients who were prescribed adalimumab or abatacept as their first biologic or not. In the final multivariate models, we adjusted for “biologic naïve” because adalimumab was more frequently prescribed as a first‐line biologic than abatacept in this dataset.

An additive model of association was used for the genetic markers (0, 1, and 2 copies). We used logistic regression to test for associations with DAS28 remission, ordinal regression for EULAR response, and linear regression for ΔDAS28. Effect sizes are reported as β coefficients with their 95% confidence intervals, and significance assessed with a *P* value, which was not adjusted for multiple testing. Calendar time was defined as the number of days elapsed between the recruitment date of each patient and the recruitment date of an index patient. The index patient was defined as the patient with the earliest recruitment date (November 23, 2001) in the subset of BRAGGSS patients included in this study. The last patient was recruited on November 6, 2020. The coding schedule is available in the Supplementary Methods. Data will be made available upon request as part of scientific collaborations.

## RESULTS

### Cohort characteristics

Following quality control, 342 BRAGGSS patients with available genetic and treatment response data were eligible for this study (Table 1). Of the 333 patients for whom adherence to MTX could be evaluated using serum levels of the drug (see Methods), 18 (5.5%) were found to be nonadherent to MTX. This real‐world, nationwide, prospective cohort of patients with RA reflects current prescribing practice in the United Kingdom: patients receiving abatacept are significantly older, have a longer disease duration, have failed biologic treatment more frequently, have more comorbidities, and do not respond as well to treatment as patients receiving adalimumab.

### The effect of genetic factors on response to treatment is consistent in adalimumab‐ or abatacept‐treated patients in BRAGGSS

EULAR response was used as the primary measure of response to treatment, as it is a validated and widely used measure of response, offering more granularity than DAS28 remission (three levels instead of two). However, because there is no universal measure of response, we conducted sensitivity analyses using DAS28 remission and ΔDAS28, a two‐level measure and a continuous measure of response, respectively.

We built a multivariate model testing for an association between the SE, drug type, their interaction, and response to treatment. Results of univariate analysis are shown in Supplementary Tables 3–5. The multivariate model was adjusted for patient demographics (age, sex, BMI) as well as disease duration, comorbidities, MTX use, presence or absence of therapeutic drug levels, and whether patients were biologic naïve. For the outcomes ΔDAS28 and DAS28 remission, we additionally adjusted for baseline DAS28.

The results of the multivariate modeling are presented in Table 2 and show no difference in the efficacy of abatacept versus adalimumab. We found weak evidence for an independent association of genetic markers with response to treatment (Val11 with EULAR response: *P* = 0.02). However, this effect was not dependent on treatment type (*P* for interaction = 0.13).

**Table 2:** Association testing of genetic factors and drug type with response to treatment in BRAGGSS*

	Remission			ΔDAS28			EULAR response		
	Coefficient (95% CI)	*P* value	FMI	Coefficient (95% CI)	*P* value	FMI	Coefficient (95% CI)	*P* value	FMI
SE									
n copies of SE	0.58 (−0.16 to 1.32)	0.13	0.005	0.33 (−0.0 to 0.66)	0.05	0.008	0.31 (−0.21 to 0.84)	0.24	0.008
Drug type	0.7 (−0.6 to 2.01)	0.29	0.004	0.36 (−0.2 to 0.92)	0.21	0.007	0.34 (−0.55 to 1.23)	0.45	0.005
SE by drug type	−0.59 (−1.47 to 0.3)	0.20	0.004	−0.24 (−0.65 to 0.17)	0.25	0.007	−0.28 (−0.94 to 0.39)	0.41	0.007
BMI	−0.04 (−0.1 to 0.01)	0.11	0.176	−0.03 (−0.05 to −0.01)	**0.02**	0.139	−0.05 (−0.09 to −0.0)	**0.03**	0.186
MTX use	0.37 (−0.42 to 1.16)	0.36	0.17	0.36 (−0.0 to 0.71)	0.05	0.154	0.56 (−0.01 to 1.14)	0.05	0.124
Biologic naïve	1.31 (0.64 to 1.98)	**1.39 × 10** ^**−4**^	0.004	0.77 (0.45 to 1.08)	**1.7 × 10** ^**−6**^	0.008	1.14 (0.62 to 1.66)	**2.00 × 10** ^**−5**^	0.005
Baseline DAS28	−0.52 (−0.83 to −0.21)	**1.2 × 10** ^**−3**^	0.014	0.61 (0.46 to 0.76)	**9.8 × 10** ^**−16**^	0.007	N/A	N/A	N/A
Sex	−0.11 (−0.8 to 0.58)	0.76	0.006	0.05 (−0.28 to 0.39)	0.75	0.007	0.04 (−0.51 to 0.59)	0.88	0.006
Age	−0.01 (−0.03 to 0.02)	0.66	0.005	−0.0 (−0.01 to 0.01)	0.81	0.009	−0.0 (−0.02 to 0.02)	0.84	0.006
Disease duration	0.01 (−0.02 to 0.04)	0.45	0.017	0.01 (0.0 to 0.03)	**0.03**	0.014	0.01 (−0.01 to 0.04)	0.25	0.017
Therapeutic drug level	1.12 (0.4 to 1.84)	**2.5 × 10** ^**−3**^	0.005	0.7 (0.38 to 1.02)	**1.55 × 10** ^**−5**^	0.012	1.13 (0.61 to 1.65)	**2.00 × 10** ^**−5**^	0.009
Comorbidity	−0.38 (−1.1 to 0.34)	0.30	0.003	0.08 (−0.25 to 0.41)	0.64	0.008	−0.11 (−0.65 to 0.42)	0.67	0.007
Val11									
n copies of Val11	0.62 (−0.1 to 1.35)	0.09	0.003	0.29 (−0.04 to 0.62)	0.08	0.045	0.63 (0.11 to 1.16)	**0.02**	0.006
Drug type	0.46 (−0.65 to 1.57)	0.42	0.004	0.21 (−0.27 to 0.69)	0.40	0.008	0.48 (−0.29 to 1.26)	0.22	0.005
Val11 by drug type	−0.47 (−1.34 to 0.4)	0.29	0.003	−0.13 (−0.54 to 0.27)	0.53	0.01	−0.51 (−1.17 to 0.15)	0.13	0.005
BMI	−0.05 (−0.1 to 0.01)	0.11	0.169	−0.03 (−0.05 to −0.01)	0.02	0.007	−0.05 (−0.09 to −0.0)	**0.03**	0.166
MTX use	0.36 (−0.43 to 1.15)	0.37	0.172	0.32 (−0.04 to 0.67)	0.08	0.139	0.52 (−0.06 to 1.1)	0.08	0.126
Biologic naïve	1.3 (0.63 to 1.98)	**1.47 × 10** ^**−4**^	0.004	0.77 (0.46 to 1.09)	**1.48 × 10** ^**−6**^	0.153	1.16 (0.64 to 1.68)	**1.00 × 10** ^**−5**^	0.006
Baseline DAS28	−0.53 (−0.84 to −0.22)	**9.5 × 10** ^**−4**^	0.013	0.6 (0.46 to 0.75)	**1.12 × 10** ^**−15**^	0.008	N/A	N/A	N/A
Sex	−0.12 (−0.82 to 0.57)	0.72	0.006	0.05 (−0.29 to 0.38)	0.79	0.007	−0.02 (−0.57 to 0.54)	0.95	0.008
Age	−0.01 (−0.03 to 0.02)	0.61	0.005	−0.0 (−0.01 to 0.01)	0.67	0.006	−0.0 (−0.02 to 0.02)	0.68	0.006
Disease duration	0.01 (−0.02 to 0.04)	0.44	0.017	0.02 (0.0 to 0.03)	**0.02**	0.008	0.01 (−0.01 to 0.04)	0.22	0.016
Therapeutic drug level	1.10 (0.37 to 1.82)	**2.9 × 10** ^**−3**^	0.005	0.69 (0.37 to 1.0)	**2.17 × 10** ^**−5**^	0.014	1.13 (0.61 to 1.65)	**2.00 × 10** ^**−5**^	0.007
Comorbidity	−0.32 (−1.04 to 0.4)	0.39	0.002	0.09 (−0.24 to 0.43)	0.58	0.011	−0.04 (−0.58 to 0.51)	0.90	0.006

* Multivariate model showing weak evidence of independent association of genetic markers with response to treatment (Val11 with EULAR response) but no difference in the efficacy of adalimumab or abatacept (no significant *P* value for the variable “drug type”). The interaction term (n copies of SE by drug type or n copies of Val11 by drug type) is not significant; therefore, the effect of genetic factors on response to treatment is similar for both drugs. BMI, body mass index; BRAGGSS, Biologics in Rheumatoid Arthritis Genetics and Genomics Study Syndicate; CI, confidence interval; DAS28, Disease Activity Score in 28 joints; FMI, fraction of missing information; MTX, methotrexate; N/A, models for EULAR response were not adjusted for baseline DAS28 because this variable is included in the definition of EULAR response (see Methods); SE, shared epitope; Val11, valine at position 11.

Response to treatment was significantly better in biologic‐naïve patients (*P* < 1.5 × 10^−4^ for any measure of response and any genetic marker tested; Table 2). Therefore, we ran the multivariate model again after stratifying patients as biologic naïve (180 patients) or not (162 patients). In the latter set of patients, we did not detect any significant difference in the efficacy of adalimumab or abatacept, genetic markers were not associated with response, and the interaction term was not significant (Supplementary Table 6). Similarly, in biologic‐naïve patients, no significant difference in the efficacy of adalimumab or abatacept was observed, but carriers of the SE or Val11 were more likely to enter remission than noncarriers (*P* = 0.03 and 0.02, respectively), and this effect was statistically significantly different between the two drugs (*P* for interaction = 0.02 for both SE and Val11), with carriers more likely to enter remission with abatacept than with adalimumab (Supplementary Table 7). However, both the effect of genetics and the interaction were absent when other measures of response to treatment (ΔDAS28; EULAR response) were used. In addition, it must be highlighted that the differential effectiveness of genetic factors on remission for the two drugs was based on small absolute patient numbers (see breakdown in Supplementary Figure 3).

Because prescribing practices have changed over time, we reran the multivariate model by adding calendar time as a covariate. Calendar time was not independently associated with any measure of response to treatment (Supplementary Table 8), and consequently, results presented in Supplementary Table 8 fully align with those presented in Table 2.

Because ACPA may be a path variable between HLA–DRB1 and treatment response, this was not adjusted for in the multivariate model. Subanalysis of ACPA‐positive patients only (n = 125) is shown in Supplementary Table 6. No association of genetic markers or drug type with response to treatment was detected, and the interaction term was not statistically significant. Therefore, we were not able to detect any differential effectiveness of abatacept over adalimumab in patients carrying one or two copies of the SE (or Val11) versus noncarriers, neither in the whole dataset nor in ACPA‐positive patients only.

## DISCUSSION

In this study of real‐world patients treated with abatacept and adalimumab, we found no significant association between treatment type and response, suggesting equivalent efficacy of the drugs. There was a significant association between the number of copies of Val11 and EULAR response (*P* = 0.02) but no significant interaction with treatment type, suggesting that weak independent effects of genetic variants on response do not significantly differ between abatacept and adalimumab. These results altogether suggest no differential responsiveness to abatacept compared to adalimumab for patients from different genetic groups (carrying either 0, 1, or 2 copies of either the SE or Val11) in this study.

We aimed to address the main limitations of previous studies by analyzing data from a large nationwide, prospective, real‐life cohort. Our approach offers a strength over previous studies through integration of drug levels in a complex statistical framework accounting for known effects, interactions, and confounding factors. Furthermore, instead of dichotomizing the SE, we incorporate the number of SE copies or the number of copies of Val11, carried by each patient. We model response to treatment in a multivariate analysis; we simultaneously test for an association of the type of treatment (abatacept or adalimumab) with response and genetic factors (number of copies of the SE or of Val11) with response and the interaction between these two variables, while adjusting for relevant covariates.

While in stratified analysis, we identified a significant differential effectiveness in carriers of SE alleles or Val11 in biologic‐naïve patients in favor of abatacept over adalimumab and regardless of ACPA status; this result requires validation in independent studies of biologic‐naïve patients. However, the results of our stratified analysis should be interpreted with caution due to important limitations: small numbers (Supplementary Figure 3), the absence of correction for multiple testing, and a lack of association with other measures of response (ΔDAS28 or EULAR response).

There is controversy in the field on the usefulness of ACPA status, ACPA titers, and/or genetic susceptibility factors in patients’ stratification for abatacept treatment, but previous studies have a number of limitations. A post hoc analysis^16^ of the first AMPLE trial conducted in biologic‐naïve patients^41^, ^42^ showed that a positive ACPA status implied a greater decrease in DAS28 over time compared to ACPA‐negative status in patients treated with abatacept or adalimumab. However, stratification of patients into treatment response categories based on ACPA titers (instead of status) was only observed with abatacept.^12^ A number of studies followed, 19 of them performed a meta‐analysis in 2020 to show an association with better response to abatacept, but not to TNFi, in ACPA‐positive patients.^10^ In 2023, an analysis of four pooled abatacept trials (AGREE, AMPLE, AVERT, and AVERT‐2) confirmed a greater efficacy of abatacept in patients with seropositive early and active RA.^11^

Genetic studies, however, have been generally conducted in smaller sample sizes, and their results are conflicting. A retrospective cohort study in 47 SE‐positive and 25 SE‐negative patients taking abatacept^15^ demonstrated a higher retention rate for SE‐positive patients. The phase 4 Early AMPLE trial^15^ reported a significantly better efficacy for abatacept than adalimumab in SE‐positive patients 24 weeks after the initiation of treatment. However, this study comprised only 80 patients in total (SE positive and SE negative across both treatment arms), and the effect was no longer present at 48 weeks. Nonetheless, patient stratification based on the number of SE copies correlated with abatacept efficacy in the TOF‐ABT study with only 140 patients in total,^13^ half taking abatacept and half taking tofacitinib. In a further small study, Inoue et al used directed acyclic graphs to identify an association between one SE allele (HLA–DRB1*04:05) and abatacept response, seemingly independent of ACPA titers.^14^ By contrast, no statistically significant difference in the efficacy of abatacept in SE‐positive patients was found in two recent large studies (>100 patients taking abatacept in each study),^17^, ^18^ although one of these studies identified a statistically significant association with Val11. Interestingly, we also found an association between copies of Val11, but not the SE, and treatment response.

Many published reports fail to fully address disease heterogeneity, which can lead to either false‐positive or false‐negative associations by failing to integrate all known demographic, clinical, serologic, and pharmacological determinants of response into a well‐powered statistical model; therefore, adjustment for potential confounders is suboptimal. In keeping with more recent studies, our study also highlights that the dichotomization of genotypes within the HLA into SE positive and SE negative might be obsolete because the strongest genetic predictors of RA susceptibility and outcome are now known to lie outside the SE.^6^, ^38^ Therefore, our study addresses many of the shortcomings of these earlier studies. In addition, the use of published cutoffs adapted to the prescribed dose of MTX may have improved the sensitivity to detect adherence.^34^

However, our study has several limitations. The sample size remains modest in comparison to genetic studies of disease susceptibility and will have limited power to detect small effect sizes when many covariates are considered to tackle issues of disease heterogeneity (eg, for stratified analysis by ACPA status). In view of prescribing practices in the United Kingdom, patients receiving abatacept compared to those receiving adalimumab differ in several respects. Patients taking abatacept tend to be older, have more comorbidities, and are less likely to be biologic naïve. Although we have attempted to adjust for these factors, we may not have fully accounted for residual confounding related to differences in patient characteristics and treatment history. Because previous studies differ widely in their study design and cohort characteristics, a meta‐analysis with our study would not be appropriate, but we think that our study design and statistical approach, including drug levels and other relevant covariates, could inform further studies, which, in the future, could be reviewed in a meta‐analysis.

Using a large real‐life, prospective, and UK‐wide cohort of patients with RA, we did not detect any difference in the efficacy between adalimumab and abatacept and only borderline significant associations with Val11 but not the SE. The weak effects of Val11 on response do not differ between drugs. Therefore, in contrast to smaller earlier studies, we were unable to detect any differential effectiveness of the two drugs in different genetic backgrounds.

## AUTHOR CONTRIBUTIONS

All authors contributed to at least one of the following manuscript preparation roles: conceptualization AND/OR methodology, software, investigation, formal analysis, data curation, visualization, and validation AND drafting or reviewing/editing the final draft. As corresponding author, Dr Viatte confirms that all authors have provided the final approval of the version to be published and takes responsibility for the affirmations regarding article submission (eg, not under consideration by another journal), the integrity of the data presented, and the statements regarding compliance with institutional review board/Declaration of Helsinki requirements.

## ROLE OF THE STUDY SPONSOR

Bristol Myers Squibb had no role in the study design or in the collection, analysis, or interpretation of the data, the writing of the manuscript, or the decision to submit the manuscript for publication. Publication of this article was not contingent upon approval by Bristol Myers Squibb.

## Supporting information


**Disclosure Form**:

**Data S1** Supporting Information
