# Supplementary material for: Comparative Effectiveness of Abatacept Versus Adalimumab in Shared Epitope Positive and Negative Patients With Rheumatoid Arthritis
Source: Arthritis Rheumatol. 2025 Sep 23;78(1):59–67. doi: 10.1002/art.43298 (PMC12854005; doi:10.1002/art.43298)
Supplement: Supplementary file 2 — Data S1 Supporting Information [file ART-78-59-s001.docx]

**Comparative Effectiveness of Abatacept versus Adalimumab in Shared Epitope positive and negative Rheumatoid Arthritis patients**

Chuan Fu Yap ^1^, Nisha Nair ^1,3^, Seema Sharma^1^, John Bowes ^1^, Amirah Binti Mohammad Ariff ^1^, Ann W Morgan^5^, John D Isaacs ^6,7^, Anthony G Wilson ^8^, Kimme L Hyrich ^2,3^, Suzan Verstappen ^2,3^, James Bluett ^1,3^, Andrew P Morris ^1,3^, Anne Barton ^1,3^, Darren Plant ^1,3,*^, Sebastien Viatte ^1,3,4,*^

^1^ Centre for Genetics and Genomics Versus Arthritis and ^2^ Centre for Epidemiology Versus Arthritis, Centre for Musculoskeletal Research, Division of Musculoskeletal and Dermatological Sciences, The University of Manchester, United Kingdom.

^3^ NIHR Manchester Biomedical Research Centre, Manchester University NHS Foundation Trust, Manchester Academic Health Science Centre, Manchester, United Kingdom.

^4^ Lydia Becker Institute of Immunology and Inflammation, Faculty of Biology, Medicine and Health, The University of Manchester, Manchester, United Kingdom.

^5^ School of Medicine University of Leeds, Leeds, UK and NIHR Leeds Biomedical Research Centre, Leeds Teaching Hospitals NHS Trust

^6^ Translational and Clinical Research Institute, Newcastle University, Newcastle-upon-Tyne, UK

^7^ Musculoskeletal Unit and NIHR Biomedical Research centre, Newcastle-upon-Tyne Hospitals NHS Foundation Trust, Newcastle-upon-Tyne, UK

^8^ School of Medicine and Medical Science, Conway Institute, University College Dublin, Dublin, Ireland

^*^ Equal contribution, joint last authors

**Supplementary files:** See next page

**Supplementary methods**

Phasing, genotype and HLA imputation:

The genotype file was phased using Eagle v2.4 and imputed on the Michigan Imputation Server (accessed on 28^th^ January 2022) with Minimac4 (34) using the Haplotype Reference Consortium (HRC) r1.1 2016 reference panel for European ancestry. HLA imputation was performed with SNP2HLA using the T1DGC reference panel (35). Imputed genotypes were filtered to remove variants with a low r^2^ score (<0.5). Custom scripts (Linux) and PLINK version 1.9 were used (36) (https://www.cog-genomics.org/plink/1.9/).

The following HLA-DRB1 alleles were considered SE alleles: *01:01, *01:02, *01:04, *01:05, *01:07, *01:08, *01:10, *01:11, *04:01, *04:04, *04:05, *04:08, *04:09, *04:10, *04:13, *04:16, *04:19, *04:21, *04:23, *04:26, *04:28, *04:29, *04:30, *04:33, *04:34, *04:35, *04:38, *04:40, *04:42, *04:43, *04:45, *10:01, *11:13, *11:26, *11:34, *14:02, *14:09, *14:13, *14:17, *14:19, *14:20, *14:21, *14:29, *14:30, *14:31, *14:32, *14:34, *14:41, *14:46, *14:47, *14:48, *04:66 and *14:06.

Coding schedule for clinical variables:

Drug Type: “adalimumab” coded as 1, abatacept coded as “0”. Biologic-naïve: “yes” coded as 1, “no” coded as 0. Remission: “DAS28<2.6” coded as 1. ΔDAS28: baselineDAS28 - follow-upDAS28. “On MTX” coded as 1, “not on MTX” coded as 0. EULAR=0 means “no response”, 1 means “intermediate” and 2 means “good response”. Presence/absence of therapeutic drug levels: 1 for “exceeding the therapeutic limit of >5 µg/ml for adalimumab, or of >10 µg/ml for abatacept”, 0 otherwise. Comorbidity: 1 for the presence of comorbidities, 0 for the absence of comorbidities.

Methotrexate (MTX) levels:

The availability of MTX drug levels in this study at each time point is presented in **Supplementary Table 2** and **Supplementary Figure 1**. Baseline MTX use (concomitant prescription) from clinical notes is documented as a categorical variable in the BRAGGSS database (onMTX variable).

Since levels were non-trough and MTX was taken weekly, levels were not expected to correlate with response. They were categorised (detected / undetected) in order to impute the missing baseline onMTX variable. False negative MTX levels can occur as samples are shipped to Manchester from 52 sites across the UK and red blood cells can still take up MTX during shipping. To palliate to this limitation in the study design, we determined MTX levels at 3 time points (before the initiation of biologic treatment and at 2 follow-up time points: 3- and 6-months) for patients with prescribed MTX, for patients with a missing record of MTX prescription (clinical notes) and 9 negative controls (patients with a variable onMTX indicating that MTX had not been prescribed).

The 9 negative controls turned out to be all negative for serum MTX (high negative predictive value (NPV) - **Supplementary Table 2**). The Sankey plot for the detection of serum methotrexate (**Supplementary Figure 1, C),** shows that, when MTX was measured, very few patients swapped from “MTX detected” to “MTX not detected” over time and vice versa. The concordance between measured MTX and the onMTX variable was 85% and the Positive Predictive Value (PPV) 100%. The association between dichotomised MTX levels and “on MTX” was highly statistically significant by logistic regression (Beta coefficient: 8.84; 95% confidence interval (0.70; 1.10); p-value: 1.3E-16).

Therefore, we used MTX levels (detectable or undetectable) to impute the missing variable onMTX (MTX status) in our dataset: where the levels were detectable for at least one time point, we decided that this patient was on MTX, when undetectable for all time points, we assumed they were not taking the drug. Consequently, the use of serum MTX levels resulted in the change from “missing” to “taking the drug” for 16 patients, and from “missing” to “not taking the drug” for 61 patients. Measured MTX levels were also used to relabel MTX use of non-adherent patients (replacing onMTX=1 by onMTX=0), where non-adherence is defined as undetectable levels of MTX in all measured timepoints. Of the 333 patients, 18 (5.5%) were deemed to be non-adherent to MTX.


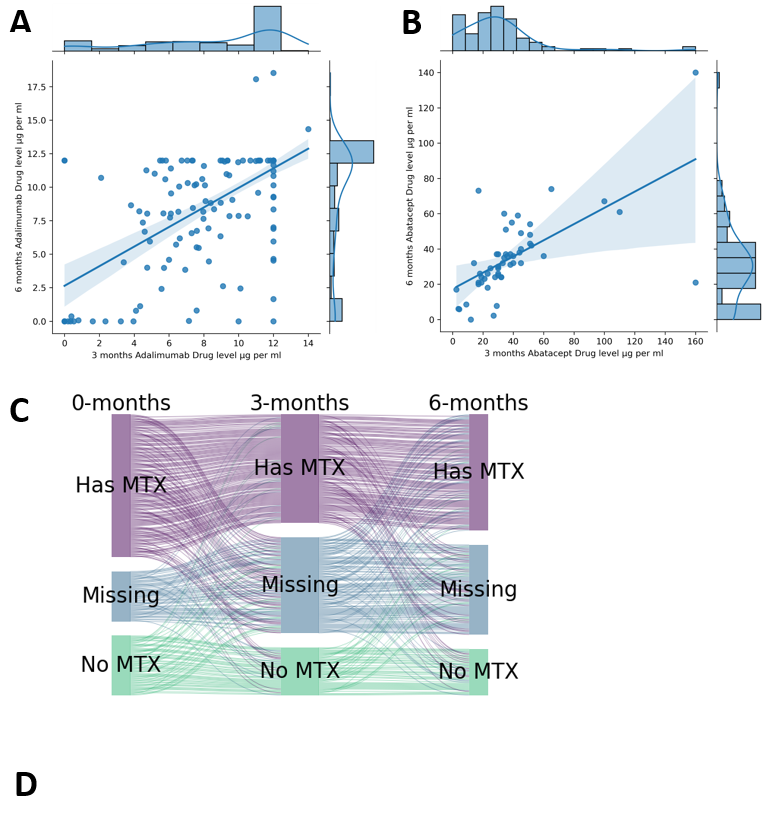


**Supplementary Figure 1: Availability and distribution of drug levels**

A) Non-trough adalimumab drug levels are well correlated between the 2 time points, but non-normally distributed, with plateau. B) The same conclusions are reached for abatacept drug levels. C) Sankey plot for the detection of serum methotrexate (MTX) at baseline (0-months), 3 months and 6 months. “Has MTX”: MTX detected in the serum. “No MTX”: MTX not detected.

|  | **At 3 months** | **At 6 months** | Correlation (3 versus 6 months) |
| --- | --- | --- | --- |
| **Adalimumab drug levels**  N available (% missing)  Concentration (µg/ml), median (IQR) | 197 (5%)  10 (5.82-12) | 184 (11%)  12 (7-12) | Spearman’s correlation:  0.65, p=1.4x10^-22^ |
| **Abatacept drug levels**  N available (% missing)  Concentration (µg/ml), median (IQR) | 94 (31%)  28.5 (16.25-36.75) | 78 (43%)  30.0 (18.5-39.5) | Spearman’s correlation:  0.73, p=8.5x10^-10^ |

**Supplementary Table 1: Availability, median levels and correlation/association between time points for biologic drug levels.** IQR: interquartile range.

|  |  | **Timepoint** | | | |
| --- | --- | --- | --- | --- | --- |
| **MTX Detected** | **onMTX** | **Baseline** | **3-months** | **6-months** | **Ever** |
| **Yes** | **Yes** | 187 | 138 | 146 | 207 (a) |
|  | **No** | 0 | 0 | 0 | 0 (c) |
|  | **Missing** | 6 | 9 | 11 | 14 |
| **No** | **Yes** | 17 | 23 | 24 | 39 (d) |
|  | **No** | 9 | 8 | 6 | 10 (b) |
|  | **Missing** | 55 | 34 | 33 | 60 |
| **Missing** | **Yes** | 17 | 60 | 51 | 102 |
|  | **No** | 44 | 45 | 47 | 49 |
|  | **Missing** | 7 | 25 | 24 | 36 |

**Supplementary Table 2: Availability of methotrexate (MTX) drug levels and concordance between detected serum levels and clinical variable**

Patient numbers at each time point where MTX was detected (Yes) or not (No), and concordance with the variable “onMTX” from clinical records (Yes: patient was prescribed MTX; no there were not). “Ever” is the total number of patients who fulfilled the criteria (f.e. MTX detected and prescribed, for the first row) at least once at any point in time. Concordance was calculated as (a+b)/(a+b+c+d)=85%. Positive Predictive Value (PPV) of measured MTX to predict onMTX: a/(a+c).

| **Covariate name** | **Beta coefficient** | **Lower CI** | **Higher CI** | **p-value** |
| --- | --- | --- | --- | --- |
| Sex | -0.27 | -0.89 | 0.34 | 0.38 |
| Age | -0.01 | -0.03 | 0.01 | 0.28 |
| Disease duration | 0.01 | -0.02 | 0.03 | 0.54 |
| Biologic-naïve | 1.45 | 0.88 | 2.01 | **5.60 x 10 ^-7^** |
| Baseline DAS28 | -0.41 | -0.68 | -0.14 | **2.8 x 10 ^-3^** |
| Comorbidity | -0.62 | -1.24 | 0.005 | 0.05 |
| MTX use | 0.62 | 0.04 | 1.21 | **0.04** |
| BMI | -0.07 | -0.12 | -0.02 | **7.5 x 10 ^-3^** |

**Supplementary Table 3: Univariate association testing of covariates with DAS28 remission**

Logistic regression was used. CI: 95% confidence interval. Beta: beta coefficient from the regression equation.

| **Covariate name** | **Beta coefficient** | **Lower CI** | **Higher CI** | **p-value** |
| --- | --- | --- | --- | --- |
| Sex | 0.08 | -0.33 | 0.48 | 0.71 |
| Age | -0.01 | -0.02 | 0.01 | 0.27 |
| Disease duration | 0.00 | -0.01 | 0.02 | 0.67 |
| Biologic-naïve | 1.07 | 0.76 | 1.39 | **8.32 x 10 ^-11^** |
| Baseline DAS28 | 0.60 | 0.43 | 0.76 | **1.04 x 10 ^-11^** |
| Comorbidity | -0.03 | -0.42 | 0.36 | 0.90 |
| MTX use | 0.49 | 0.12 | 0.85 | **8.7 x 10 ^-3^** |
| BMI | -0.03 | -0.06 | 0.00 | **0.03** |

**Supplementary Table 4: Univariate association testing of covariates with ΔDAS28**

Linear regression was used. CI: 95% confidence interval. Beta: beta coefficient from the regression equation.

| **Covariate name** | **Beta coefficient** | **Lower CI** | **Higher CI** | **p-value** |
| --- | --- | --- | --- | --- |
| Sex | -0.02 | -0.53 | 0.49 | 0.94 |
| Age | -0.01 | -0.03 | 0.01 | 0.26 |
| Disease duration | 0.00 | -0.02 | 0.02 | 0.74 |
| Biologic-naïve | 1.41 | 0.96 | 1.85 | **5.27 x 10 ^-10^** |
| Baseline DAS28 | 0.00 | -0.23 | 0.23 | 1.00 |
| Comorbidity | -0.36 | -0.83 | 0.11 | 0.14 |
| MTX use | 0.69 | 0.24 | 1.15 | **2.86 x 10 ^-3^** |
| BMI | -0.05 | -0.09 | -0.02 | **4.65 x 10 ^-3^** |

**Supplementary Table 5: Univariate association testing of covariates with EULAR response**

Ordinal regression was used. CI: 95% confidence interval. Beta: beta coefficient from the regression equation.


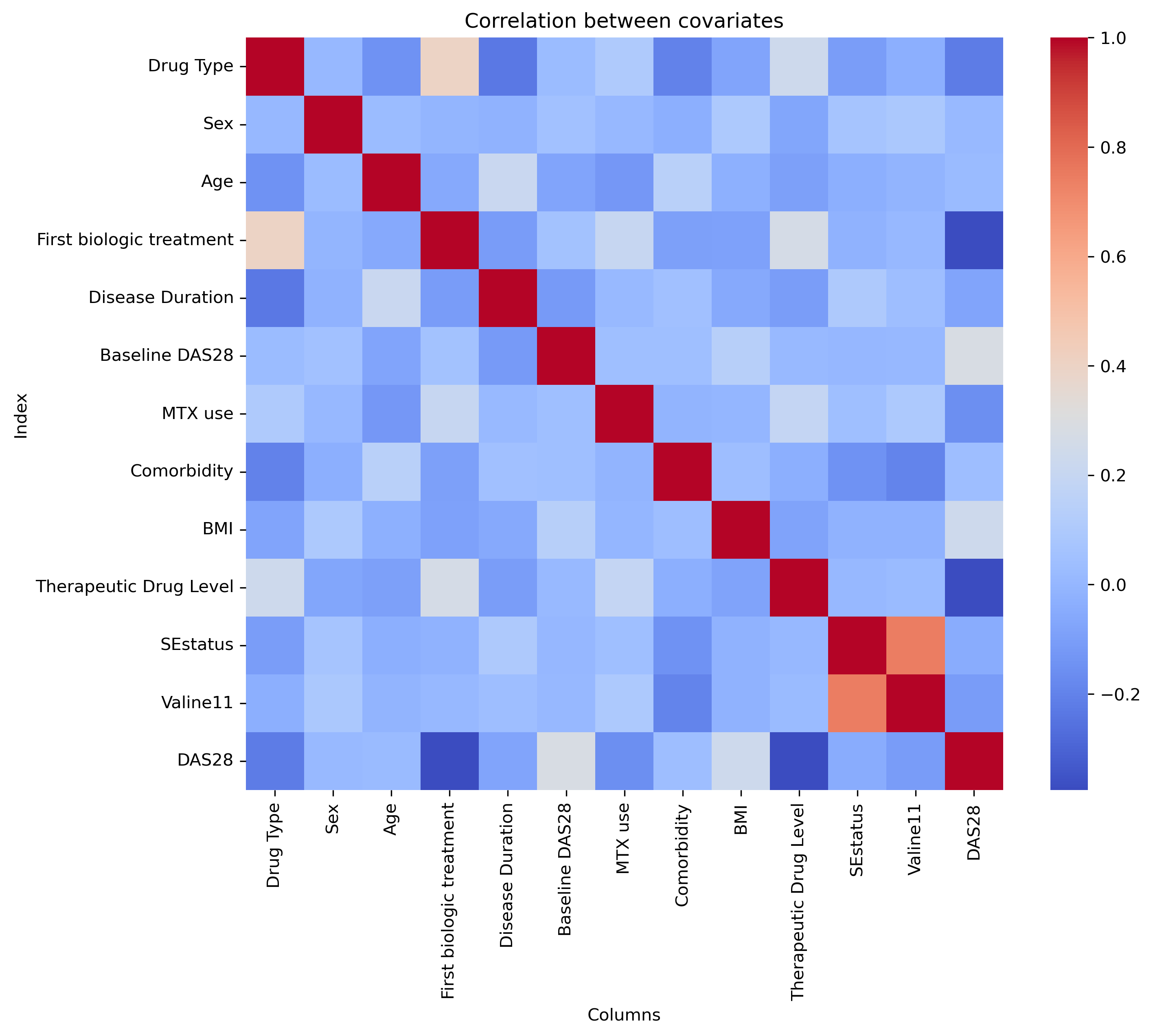


**Supplementary Figure 2: Correlation matrix heatmap**

Covariates show a low level of correlation (Pairwise Spearman correlation coefficients).

|  |  | **Remission** | | | **ΔDAS28** | | | **EULAR response** | | |
| --- | --- | --- | --- | --- | --- | --- | --- | --- | --- | --- |
|  |  | **Coef. (95% CI interval)** | **p-value** | **FMI** | **Coef. (95% CI interval)** | **p-value** | **FMI** | **Coef. (95% CI interval)** | **p-value** | **FMI** |
| **SE** | N copies of SE | 0.06 (-0.88;1.0) | 0.90 | 0.008 | 0.25 (-0.16;0.66) | 0.24 | 0.007 | 0.18 (-0.45;0.8) | 0.58 | 0.009 |
|  | Drug type | -0.78 (-3.04;1.49) | 0.50 | 0.006 | 0.42 (-0.41;1.24) | 0.32 | 0.007 | 0.05 (-1.26;1.35) | 0.94 | 0.006 |
|  | SE * Drug type | 0.82 (-0.8;2.44) | 0.32 | 0.012 | -0.1 (-0.77;0.57) | 0.78 | 0.011 | 0.07 (-0.99;1.13) | 0.90 | 0.009 |
|  | BMI | 0.01 (-0.09;0.1) | 0.90 | 0.206 | -0.02 (-0.06;0.02) | 0.27 | 0.142 | -0.04 (-0.1;0.02) | 0.16 | 0.179 |
|  | MTX use | 0.65 (-0.72;2.01) | 0.35 | 0.185 | 0.61 (0.1;1.13) | **0.02** | 0.148 | 0.89 (0.11;1.68) | **0.03** | 0.126 |
|  | Baseline DAS28 | -0.41 (-0.93;0.1) | 0.12 | 0.016 | 0.64 (0.41;0.86) | **2.8 x 10^-8^** | 0.014 | N/A | N/A | N/A |
|  | Sex | 0.04 (-1.23;1.31) | 0.95 | 0.009 | 0.25 (-0.3;0.79) | 0.38 | 0.012 | 0.21 (-0.64;1.05) | 0.63 | 0.013 |
|  | Age | -0.0 (-0.05;0.04) | 0.85 | 0.006 | 0.0 (-0.02;0.02) | 0.81 | 0.015 | 0.0 (-0.03;0.03) | 0.94 | 0.012 |
|  | Disease Duration | 0.02 (-0.04;0.07) | 0.55 | 0.015 | 0.01 (-0.01;0.03) | 0.28 | 0.021 | 0.01 (-0.03;0.04) | 0.67 | 0.031 |
|  | Therapeutic Drug Level | 1.08 (-0.06;2.21) | 0.06 | 0.004 | 0.72 (0.27;1.17) | **1.7 x 10^-3^** | 0.01 | 1.27 (0.56;1.99) | **4.9 x 10^-4^** | 0.009 |
|  | Comorbidity | 0.04 (-1.19;1.27) | 0.95 | 0.007 | 0.21 (-0.31;0.73) | 0.43 | 0.012 | 0.11 (-0.66;0.89) | 0.77 | 0.011 |
| **Val11** | N copies of Val11 | -0.08 (-1.03;0.87) | 0.87 | 0.01 | 0.19 (-0.23;0.61) | 0.37 | 0.013 | 0.59 (-0.06;1.24) | 0.08 | 0.019 |
|  | Drug type | -0.3 (-2.04;1.43) | 0.73 | 0.004 | 0.25 (-0.44;0.94) | 0.48 | 0.009 | 0.24 (-0.87;1.35) | 0.67 | 0.009 |
|  | Val11*Drug type | 0.52 (-0.97;2.02) | 0.49 | 0.003 | 0.08 (-0.59;0.75) | 0.81 | 0.007 | -0.1 (-1.13;0.93) | 0.84 | 0.01 |
|  | BMI | 0.0 (-0.09;0.09) | 0.99 | 0.204 | -0.02 (-0.06;0.01) | 0.23 | 0.149 | -0.05 (-0.11;0.01) | 0.12 | 0.248 |
|  | MTX use | 0.62 (-0.74;1.99) | 0.37 | 0.19 | 0.56 (0.03;1.08) | **0.04** | 0.148 | 0.76 (-0.06;1.58) | 0.07 | 0.168 |
|  | Baseline DAS28 | -0.47 (-0.96;0.03) | 0.07 | 0.015 | 0.63 (0.41;0.86) | **3.05 x 10^-8^** | 0.012 | N/A | N/A | N/A |
|  | Sex | 0.11 (-1.15;1.37) | 0.87 | 0.009 | 0.22 (-0.32;0.77) | 0.43 | 0.011 | 0.09 (-0.77;0.95) | 0.84 | 0.009 |
|  | Age | -0.0 (-0.05;0.05) | 0.93 | 0.005 | 0.0 (-0.02;0.02) | 1.00 | 0.016 | -0.0 (-0.04;0.03) | 0.76 | 0.015 |
|  | Disease Duration | 0.02 (-0.04;0.07) | 0.54 | 0.016 | 0.01 (-0.01;0.04) | 0.20 | 0.021 | 0.01 (-0.02;0.04) | 0.56 | 0.037 |
|  | Therapeutic Drug Level | 1.1 (-0.03;2.23) | 0.06 | 0.004 | 0.71 (0.26;1.17) | **2 x 10^-3^** | 0.009 | 1.28 (0.56;2.0) | **5 x 10^-4^** | 0.008 |
|  | Comorbidity | -0.01 (-1.27;1.24) | 0.98 | 0.008 | 0.23 (-0.3;0.75) | 0.40 | 0.013 | 0.27 (-0.52;1.06) | 0.50 | 0.01 |

**Supplementary Table 6: Association testing of genetic factors and drug type with response to treatment, restricted to patients starting a second line (or higher order) biologic treatment (total number of non-biologic-naïve patients: 162).** FMI stands for fraction of missing information. This analysis complements Table 2, see legend for further information. P-values below the nominal significance level of 0.05 (no correction for multiple testing) are highlighted in bold. No difference in the efficacy of adalimumab or abatacept is observed. The interaction term (N copies of SE *Drug type or N copies of Val11*Drug type) is not significant, therefore the effect of genetic factors on response to treatment is similar for both drugs. N/A: models for EULAR response were not adjusted for Baseline DAS28, as this variable is included in the definition of EULAR response (see methods). N: number.

|  |  | **Remission** | | | **ΔDAS28** | | | **EULAR response** | | |
| --- | --- | --- | --- | --- | --- | --- | --- | --- | --- | --- |
|  |  | **Coef. (95% CI interval)** | **p-value** | **FMI** | **Coef. (95% CI interval)** | **p-value** | **FMI** | **Coef. (95% CI interval)** | **p-value** | **FMI** |
| **SE** | N copies of SE | 1.64 (0.14;3.14) | **0.03** | 0.01 | 0.53 (-0.09;1.15) | 0.10 | 0.016 | 0.74 (-0.29;1.77) | 0.16 | 0.007 |
|  | Drug type | 2.17 (-0.27;4.61) | 0.08 | 0.007 | 0.35 (-0.61;1.31) | 0.47 | 0.014 | 0.86 (-0.62;2.33) | 0.25 | 0.007 |
|  | SE * Drug type | -1.85 (-3.47;-0.24) | **0.02** | 0.009 | -0.49 (-1.18;0.2) | 0.16 | 0.013 | -0.81 (-1.94;0.33) | 0.16 | 0.005 |
|  | BMI | -0.08 (-0.15;-0.0) | **0.04** | 0.143 | -0.04 (-0.07;-0.01) | **0.02** | 0.119 | -0.05 (-0.1;0.01) | 0.12 | 0.129 |
|  | MTX use | 0.18 (-0.83;1.19) | 0.73 | 0.112 | 0.09 (-0.43;0.62) | 0.72 | 0.125 | 0.17 (-0.76;1.1) | 0.72 | 0.119 |
|  | Baseline DAS28 | -0.47 (-0.9;-0.04) | **0.03** | 0.015 | 0.63 (0.42;0.85) | **5.75 x 10^-9^** | 0.01 | N/A | N/A | N/A |
|  | Sex | -0.18 (-1.03;0.67) | 0.68 | 0.01 | -0.02 (-0.46;0.41) | 0.91 | 0.007 | -0.02 (-0.77;0.73) | 0.95 | 0.005 |
|  | Age | -0.01 (-0.04;0.02) | 0.72 | 0.006 | -0.0 (-0.02;0.01) | 0.74 | 0.009 | -0.0 (-0.03;0.02) | 0.82 | 0.008 |
|  | Disease Duration | 0.01 (-0.03;0.04) | 0.68 | 0.023 | 0.02 (-0.0;0.04) | 0.08 | 0.015 | 0.02 (-0.02;0.05) | 0.35 | 0.014 |
|  | Therapeutic Drug Level | 1.32 (0.29;2.35) | **0.01** | 0.009 | 0.76 (0.29;1.23) | **1.5 x 10^-3^** | 0.015 | 1.13 (0.32;1.94) | **6.46 x 10^-3^** | 0.009 |
|  | Comorbidity | -0.66 (-1.59;0.26) | 0.16 | 0.004 | -0.02 (-0.46;0.43) | 0.94 | 0.005 | -0.29 (-1.06;0.48) | 0.46 | 0.003 |
| **Val11** | N copies of Val11 | 2.17 (0.42;3.92) | **0.02** | 0.006 | 0.41 (-0.22;1.04) | 0.20 | 0.013 | 1.01 (-0.15;2.17) | 0.09 | 0.004 |
|  | Drug type | 2.08 (-0.23;4.38) | 0.08 | 0.005 | 0.07 (-0.76;0.89) | 0.88 | 0.014 | 0.81 (-0.52;2.13) | 0.23 | 0.006 |
|  | Val11*Drug type | -2.1 (-3.94;-0.26) | **0.02** | 0.006 | -0.3 (-0.99;0.38) | 0.38 | 0.011 | -1.0 (-2.24;0.24) | 0.11 | 0.003 |
|  | BMI | -0.08 (-0.15;-0.0) | **0.04** | 0.138 | -0.04 (-0.07;-0.0) | **0.03** | 0.113 | -0.04 (-0.1;0.02) | 0.15 | 0.118 |
|  | MTX use | 0.38 (-0.66;1.43) | 0.47 | 0.12 | 0.09 (-0.43;0.62) | 0.72 | 0.111 | 0.18 (-0.75;1.12) | 0.70 | 0.117 |
|  | Baseline DAS28 | -0.47 (-0.9;-0.05) | **0.03** | 0.014 | 0.63 (0.42;0.84) | **6.21 x 10^-9^** | 0.01 | N/A | N/A | N/A |
|  | Sex | -0.21 (-1.07;0.64) | 0.63 | 0.01 | -0.02 (-0.46;0.41) | 0.92 | 0.008 | -0.06 (-0.81;0.7) | 0.88 | 0.005 |
|  | Age | -0.0 (-0.03;0.03) | 0.77 | 0.007 | -0.0 (-0.02;0.01) | 0.71 | 0.007 | -0.0 (-0.03;0.02) | 0.82 | 0.006 |
|  | Disease Duration | 0.01 (-0.03;0.04) | 0.77 | 0.021 | 0.02 (-0.0;0.04) | 0.07 | 0.013 | 0.02 (-0.02;0.05) | 0.34 | 0.015 |
|  | Therapeutic Drug Level | 1.16 (0.13;2.19) | **0.03** | 0.008 | 0.72 (0.25;1.18) | **2.6 x 10^-3^** | 0.012 | 1.08 (0.27;1.88) | **8.79 x 10^-3^** | 0.008 |
|  | Comorbidity | -0.5 (-1.42;0.43) | 0.29 | 0.004 | -0.01 (-0.46;0.43) | 0.96 | 0.005 | -0.27 (-1.04;0.5) | 0.50 | 0.003 |

**Supplementary Table 7: Association testing of genetic factors and drug type with response to treatment, restricted to biologic-naïve patients (total patient number: 180).** FMI stands for fraction of missing information. This analysis complements Table 2, see legend for further information. P-values below the nominal significance level of 0.05 (no correction for multiple testing) are highlighted in bold. No difference in the efficacy of adalimumab or abatacept is observed. Carriers of the SE or Val11 are more likely to enter remission than non-carriers, and this effect is statistically significantly different between the two drugs, with carriers significantly more likely to enter remission when treated with abatacept than when treated with adalimumab (the interaction term (N copies of SE *Drug type or N copies of Val11*Drug type) is significant, therefore there is a statistically significant differential effectiveness of genetic factors on remission for the two drugs in biologic-naïve patients). However, this analysis is based on very small absolute patient numbers (see breakdown in **Supplementary Figure 3**).

A)


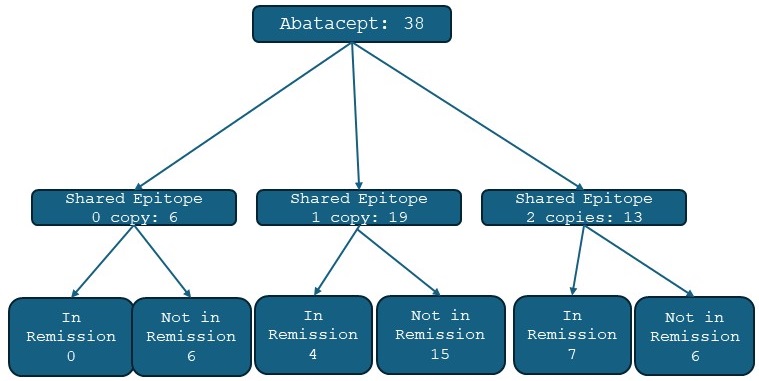


B)


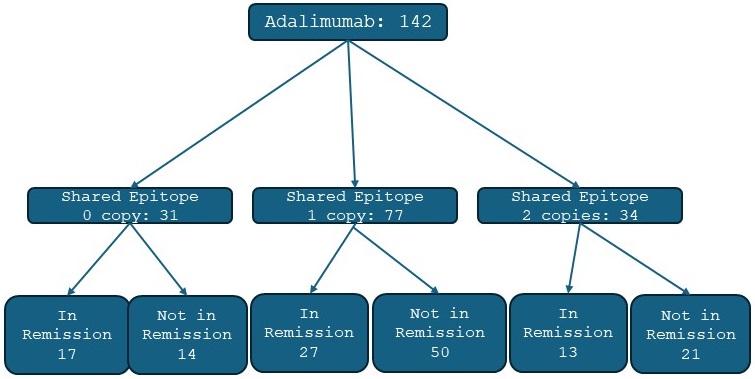


**Supplementary Figure 3: Breakdown of the 180 biologic-naïve patients analysed in Supplementary Table 7.**

38 patients received abatacept as first line biologic drug (A), while 142 received adalimumab as first line choice. Numbers in each box refer to number of patients within the parental category satisfying the condition outlined in the box and therefore included in the analysis presented in Supplementary Table 7.

|  |  | **Remission** | | | **ΔDAS28** | | | **EULAR response** | | |
| --- | --- | --- | --- | --- | --- | --- | --- | --- | --- | --- |
|  |  | **Coef. (95% CI interval)** | **p-value** | **FMI** | **Coef. (95% CI interval)** | **p-value** | **FMI** | **Coef. (95% CI interval)** | **p-value** | **FMI** |
| **SE** | N copies of SE | 0.58 (-0.17;1.32) | 0.13 | 0.006 | 0.33 (-0.0;0.66) | 0.05 | 0.007 | 0.32 (-0.2;0.84) | 0.23 | 0.006 |
|  | Drug type | 0.65 (-0.72;2.03) | 0.35 | 0.006 | 0.36 (-0.22;0.95) | 0.22 | 0.008 | 0.41 (-0.52;1.35) | 0.39 | 0.005 |
|  | SE * Drug type | -0.59 (-1.47;0.3) | 0.20 | 0.004 | -0.24 (-0.65;0.17) | 0.25 | 0.008 | -0.28 (-0.94;0.38) | 0.41 | 0.005 |
|  | BMI | -0.05 (-0.1;0.01) | 0.11 | 0.191 | -0.03 (-0.05;-0.0) | **0.02** | 0.155 | -0.05 (-0.09;-0.01) | **0.03** | 0.188 |
|  | MTX use | 0.35 (-0.43;1.14) | 0.38 | 0.168 | 0.37 (0.01;0.73) | **0.04** | 0.166 | 0.57 (-0.02;1.16) | 0.06 | 0.156 |
|  | Biologic-naïve | 1.3 (0.61;1.98) | **2 x 10^-4^** | 0.005 | 0.77 (0.45;1.09) | **2.41 x 10^-6^** | 0.009 | 1.15 (0.63;1.68) | **2.00 x 10^-5^** | 0.007 |
|  | Baseline DAS28 | -0.52 (-0.83;-0.2) | **1.2 x 10^-3^** | 0.011 | 0.61 (0.46;0.76) | **1.88 x 10^-15^** | 0.008 | N/A | N/A | N/A |
|  | Sex | -0.11 (-0.8;0.59) | 0.76 | 0.01 | 0.06 (-0.27;0.39) | 0.73 | 0.006 | 0.05 (-0.5;0.6) | 0.86 | 0.008 |
|  | Age | -0.01 (-0.03;0.02) | 0.67 | 0.006 | -0.0 (-0.01;0.01) | 0.82 | 0.006 | -0.0 (-0.02;0.02) | 0.83 | 0.006 |
|  | Disease Duration | 0.01 (-0.02;0.04) | 0.46 | 0.019 | 0.01 (0.0;0.03) | **0.03** | 0.019 | 0.01 (-0.01;0.04) | 0.26 | 0.021 |
|  | Therapeutic Drug Level | 1.11 (0.38;1.84) | **2.9 x 10^-3^** | 0.007 | 0.7 (0.38;1.02) | **1.64 x 10^-5^** | 0.009 | 1.14 (0.62;1.67) | **2.00 x 10^-5^** | 0.01 |
|  | Calendar Date | -0.0 (-0.0;0.0) | 0.83 | 0.005 | 0.0 (-0.0;0.0) | 0.96 | 0.01 | 0.0 (-0.0;0.0) | 0.67 | 0.007 |
|  | Comorbidity | -0.37 (-1.09;0.35) | 0.32 | 0.004 | 0.08 (-0.26;0.41) | 0.65 | 0.007 | -0.12 (-0.66;0.41) | 0.65 | 0.006 |
| **Val11** | N copies of Val11 | 0.62 (-0.1;1.35) | 0.09 | 0.002 | 0.29 (-0.04;0.62) | 0.08 | 0.007 | 0.66 (0.12;1.19) | **0.02** | 0.006 |
|  | Drug type | 0.43 (-0.77;1.64) | 0.48 | 0.005 | 0.2 (-0.28;0.68) | 0.41 | 0.009 | 0.59 (-0.25;1.44) | 0.17 | 0.006 |
|  | Val11*Drug type | -0.47 (-1.34;0.4) | 0.29 | 0.002 | -0.13 (-0.53;0.28) | 0.54 | 0.006 | -0.52 (-1.19;0.14) | 0.12 | 0.006 |
|  | BMI | -0.05 (-0.1;0.01) | 0.10 | 0.185 | -0.03 (-0.05;-0.0) | **0.02** | 0.153 | -0.05 (-0.09;-0.01) | **0.03** | 0.166 |
|  | MTX use | 0.34 (-0.45;1.13) | 0.39 | 0.171 | 0.33 (-0.03;0.69) | 0.07 | 0.166 | 0.54 (-0.05;1.12) | 0.07 | 0.154 |
|  | Biologic-naïve | 1.3 (0.61;1.98) | **2 x 10^-4^** | 0.005 | 0.77 (0.46;1.09) | **1.47 x 10^-6^** | 0.009 | 1.19 (0.66;1.71) | **1.00 x 10^-5^** | 0.006 |
|  | Baseline DAS28 | -0.53 (-0.84;-0.21) | **1 x 10^-3^** | 0.01 | 0.6 (0.45;0.75) | **1.48x 10^-15^** | 0.007 | N/A | N/A | N/A |
|  | Sex | -0.12 (-0.81;0.57) | 0.73 | 0.01 | 0.05 (-0.28;0.38) | 0.77 | 0.005 | -0.01 (-0.56;0.54) | 0.97 | 0.008 |
|  | Age | -0.01 (-0.03;0.02) | 0.62 | 0.006 | -0.0 (-0.01;0.01) | 0.68 | 0.006 | -0.0 (-0.02;0.02) | 0.67 | 0.007 |
|  | Disease Duration | 0.01 (-0.02;0.04) | 0.45 | 0.019 | 0.02 (0.0;0.03) | **0.02** | 0.019 | 0.01 (-0.01;0.04) | 0.22 | 0.017 |
|  | Therapeutic Drug Level | 1.09 (0.36;1.82) | **3.4 x 10^-3^** | 0.007 | 0.69 (0.37;1.0) | **2.07 x 10^-5^** | 0.009 | 1.14 (0.62;1.66) | **2.00 x 10^-5^** | 0.006 |
|  | Calendar Date | -0.0 (-0.0;0.0) | 0.90 | 0.004 | 0.09 (-0.24;0.43) | 0.58 | 0.006 | 0.0 (-0.0;0.0) | 0.49 | 0.008 |
|  | Comorbidity | -0.31 (-1.04;0.41) | 0.40 | 0.004 | 0.29 (-0.04;0.62) | 0.08 | 0.007 | -0.06 (-0.6;0.48) | 0.83 | 0.005 |

**Supplementary Table 8: Association testing of genetic factors and drug type with response to treatment adjusted for calendar date.**

FMI stands for fraction of missing information. This analysis complements Table 2, see legend for further information. P-values below the nominal significance level of 0.05 (no correction for multiple testing) are highlighted in bold. Calendar date is not significantly associated with any measure of response to treatment. Therefore, results presented in this table align with those presented in Table 2: the multivariate model shows an independent association between the carriage of Val11 and EULAR response, but no evidence of independent association of drug type or other genetic markers with other measures of response to treatment.

|  |  | **Remission** | | | **ΔDAS28** | | | **EULAR response** | | |
| --- | --- | --- | --- | --- | --- | --- | --- | --- | --- | --- |
|  |  | **Coef. (95% CI interval)** | **p-value** | **FMI** | **Coef. (95% CI interval)** | **p-value** | **FMI** | **Coef. (95% CI interval)** | **p-value** | **FMI** |
| **SE** | N copies of SE | -1.03 (-3.15;1.1) | 0.34 | 0.005 | -0.96 (-1.99;0.08) | 0.07 | 0.009 | -1.56 (-3.45;0.34) | 0.11 | 0.012 |
|  | Drug type | -1.53 (-5.34;2.28) | 0.43 | 0.007 | -0.82 (-2.6;0.96) | 0.37 | 0.01 | -2.92 (-6.25;0.41) | 0.09 | 0.01 |
|  | SE*Drug type | 0.85 (-1.36;3.06) | 0.45 | 0.006 | 0.96 (-0.12;2.03) | 0.08 | 0.009 | 1.83 (-0.19;3.84) | 0.08 | 0.011 |
|  | BMI | -0.01 (-0.1;0.08) | 0.84 | 0.104 | -0.02 (-0.06;0.03) | 0.48 | 0.095 | -0.04 (-0.12;0.04) | 0.37 | 0.089 |
|  | MTX use | 0.57 (-0.94;2.08) | 0.46 | 0.142 | 0.39 (-0.27;1.04) | 0.25 | 0.057 | 0.87 (-0.3;2.04) | 0.14 | 0.049 |
|  | Biologic-naïve | 1.25 (-0.14;2.65) | 0.08 | 0.004 | 0.33 (-0.28;0.95) | 0.29 | 0.004 | 1.31 (0.22;2.41) | **0.02** | 0.005 |
|  | Baseline DAS28 | -0.58 (-1.1;-0.05) | **0.03** | 0.003 | 0.71 (0.48;0.94) | **1.55 x 10 ^-9^** | 0.004 | N/A | N/A | N/A |
|  | Sex | 0.67 (-0.41;1.74) | 0.22 | 0.004 | 0.19 (-0.3;0.69) | 0.45 | 0.006 | 0.11 (-0.83;1.06) | 0.81 | 0.009 |
|  | Age | 0.0 (-0.04;0.04) | 0.96 | 0.009 | 0.01 (-0.01;0.03) | 0.38 | 0.003 | 0.02 (-0.02;0.05) | 0.40 | 0.003 |
|  | Disease Duration | 0.02 (-0.03;0.07) | 0.36 | 0.013 | 0.03 (0.0;0.05) | **0.03** | 0.026 | 0.03 (-0.02;0.08) | 0.26 | 0.041 |
|  | Drug Therapeutic Level | 1.22 (-0.13;2.56) | 0.08 | 0.003 | 1.05 (0.5;1.61) | **2 x 10 ^-4^** | 0.005 | 1.63 (0.57;2.7) | **2.69 x 10 ^-3^** | 0.003 |
|  | Comorbidity | -0.94 (-2.58;0.7) | 0.26 | 0.001 | -0.46 (-1.2;0.29) | 0.23 | 0.004 | -1.2 (-2.64;0.24) | 0.10 | 0.004 |
| **Val11** | N copies of Val11 | -0.0 (-2.27;2.26) | 1.00 | 0.002 | -0.46 (-1.39;0.48) | 0.34 | 0.003 | 0.06 (-1.58;1.71) | 0.94 | 0.004 |
|  | Drug type | -0.15 (-3.8;3.5) | 0.94 | 0.004 | -0.14 (-1.69;1.4) | 0.86 | 0.005 | -0.43 (-3.19;2.33) | 0.76 | 0.004 |
|  | Val11*Drug type | 0.12 (-2.23;2.46) | 0.92 | 0.002 | 0.62 (-0.37;1.61) | 0.22 | 0.003 | 0.36 (-1.4;2.12) | 0.69 | 0.004 |
|  | BMI | -0.01 (-0.1;0.08) | 0.89 | 0.106 | -0.01 (-0.06;0.03) | 0.59 | 0.094 | -0.03 (-0.11;0.06) | 0.50 | 0.118 |
|  | MTX use | 0.6 (-0.9;2.11) | 0.43 | 0.158 | 0.39 (-0.27;1.06) | 0.27 | 0.084 | 0.76 (-0.43;1.96) | 0.21 | 0.084 |
|  | Biologic-naïve | 1.21 (-0.18;2.61) | 0.09 | 0.004 | 0.32 (-0.3;0.95) | 0.31 | 0.004 | 1.16 (0.06;2.26) | **0.04** | 0.005 |
|  | Baseline DAS28 | -0.58 (-1.1;-0.06) | **0.03** | 0.003 | 0.69 (0.46;0.93) | **5.92 x 10^-9^** | 0.004 | N/A | N/A | N/A |
|  | Sex | 0.7 (-0.37;1.77) | 0.20 | 0.004 | 0.2 (-0.3;0.7) | 0.44 | 0.006 | 0.05 (-0.88;0.99) | 0.91 | 0.004 |
|  | Age | 0.01 (-0.04;0.05) | 0.80 | 0.008 | 0.01 (-0.01;0.03) | 0.24 | 0.004 | 0.02 (-0.02;0.06) | 0.41 | 0.005 |
|  | Disease Duration | 0.02 (-0.03;0.07) | 0.36 | 0.013 | 0.03 (0.0;0.05) | **0.04** | 0.026 | 0.02 (-0.02;0.07) | 0.31 | 0.034 |
|  | Drug Therapeutic Level | 1.18 (-0.16;2.52) | 0.08 | 0.003 | 1.02 (0.46;1.58) | **4 x 10^-4^** | 0.004 | 1.6 (0.55;2.66) | **2.98 x 10 ^-3^** | 0.003 |
|  | Comorbidity | -0.63 (-2.27;1.01) | 0.45 | 0.002 | -0.31 (-1.08;0.46) | 0.43 | 0.005 | -0.7 (-2.2;0.79) | 0.36 | 0.005 |

**Supplementary Table 9: Association testing of genetic factors and drug type with response to treatment in ACPA-positive patients (n=125).**

FMI stands for fraction of missing information. This analysis complements Table 2, see legend for further information. P-values below the nominal significance level of 0.05 (no correction for multiple testing) are highlighted in bold. Multivariate model shows no evidence of independent association of genetic markers or drug type with response to treatment.

**Comparative Effectiveness of Abatacept versus Adalimumab in Shared Epitope positive and negative Rheumatoid Arthritis patients**

Chuan Fu Yap ^1^, Nisha Nair ^1,3^, Seema Sharma^1^, John Bowes ^1^, Amirah Binti Mohammad Ariff ^1^, Ann W Morgan^5^, John D Isaacs ^6,7^, Anthony G Wilson ^8^, Kimme L Hyrich ^2,3^, Suzan Verstappen ^2,3^, James Bluett ^1,3^, Andrew P Morris ^1,3^, Anne Barton ^1,3^, Darren Plant ^1,3,*^, Sebastien Viatte ^1,3,4,*^

^1^ Centre for Genetics and Genomics Versus Arthritis and ^2^ Centre for Epidemiology Versus Arthritis, Centre for Musculoskeletal Research, Division of Musculoskeletal and Dermatological Sciences, The University of Manchester, United Kingdom.

^3^ NIHR Manchester Biomedical Research Centre, Manchester University NHS Foundation Trust, Manchester Academic Health Science Centre, Manchester, United Kingdom.

^4^ Lydia Becker Institute of Immunology and Inflammation, Faculty of Biology, Medicine and Health, The University of Manchester, Manchester, United Kingdom.

^5^ School of Medicine University of Leeds, Leeds, UK and NIHR Leeds Biomedical Research Centre, Leeds Teaching Hospitals NHS Trust

^6^ Translational and Clinical Research Institute, Newcastle University, Newcastle-upon-Tyne, UK

^7^ Musculoskeletal Unit and NIHR Biomedical Research centre, Newcastle-upon-Tyne Hospitals NHS Foundation Trust, Newcastle-upon-Tyne, UK

^8^ School of Medicine and Medical Science, Conway Institute, University College Dublin, Dublin, Ireland

^*^ Equal contribution, joint last authors

**Supplementary files:** See next page

**Supplementary methods**

Phasing, genotype and HLA imputation:

The genotype file was phased using Eagle v2.4 and imputed on the Michigan Imputation Server (accessed on 28^th^ January 2022) with Minimac4 (34) using the Haplotype Reference Consortium (HRC) r1.1 2016 reference panel for European ancestry. HLA imputation was performed with SNP2HLA using the T1DGC reference panel (35). Imputed genotypes were filtered to remove variants with a low r^2^ score (<0.5). Custom scripts (Linux) and PLINK version 1.9 were used (36) (https://www.cog-genomics.org/plink/1.9/).

The following HLA-DRB1 alleles were considered SE alleles: *01:01, *01:02, *01:04, *01:05, *01:07, *01:08, *01:10, *01:11, *04:01, *04:04, *04:05, *04:08, *04:09, *04:10, *04:13, *04:16, *04:19, *04:21, *04:23, *04:26, *04:28, *04:29, *04:30, *04:33, *04:34, *04:35, *04:38, *04:40, *04:42, *04:43, *04:45, *10:01, *11:13, *11:26, *11:34, *14:02, *14:09, *14:13, *14:17, *14:19, *14:20, *14:21, *14:29, *14:30, *14:31, *14:32, *14:34, *14:41, *14:46, *14:47, *14:48, *04:66 and *14:06.

Coding schedule for clinical variables:

Drug Type: “adalimumab” coded as 1, abatacept coded as “0”. Biologic-naïve: “yes” coded as 1, “no” coded as 0. Remission: “DAS28<2.6” coded as 1. ΔDAS28: baselineDAS28 - follow-upDAS28. “On MTX” coded as 1, “not on MTX” coded as 0. EULAR=0 means “no response”, 1 means “intermediate” and 2 means “good response”. Presence/absence of therapeutic drug levels: 1 for “exceeding the therapeutic limit of >5 µg/ml for adalimumab, or of >10 µg/ml for abatacept”, 0 otherwise. Comorbidity: 1 for the presence of comorbidities, 0 for the absence of comorbidities.

Methotrexate (MTX) levels:

The availability of MTX drug levels in this study at each time point is presented in **Supplementary Table 2** and **Supplementary Figure 1**. Baseline MTX use (concomitant prescription) from clinical notes is documented as a categorical variable in the BRAGGSS database (onMTX variable).

Since levels were non-trough and MTX was taken weekly, levels were not expected to correlate with response. They were categorised (detected / undetected) in order to impute the missing baseline onMTX variable. False negative MTX levels can occur as samples are shipped to Manchester from 52 sites across the UK and red blood cells can still take up MTX during shipping. To palliate to this limitation in the study design, we determined MTX levels at 3 time points (before the initiation of biologic treatment and at 2 follow-up time points: 3- and 6-months) for patients with prescribed MTX, for patients with a missing record of MTX prescription (clinical notes) and 9 negative controls (patients with a variable onMTX indicating that MTX had not been prescribed).

The 9 negative controls turned out to be all negative for serum MTX (high negative predictive value (NPV) - **Supplementary Table 2**). The Sankey plot for the detection of serum methotrexate (**Supplementary Figure 1, C),** shows that, when MTX was measured, very few patients swapped from “MTX detected” to “MTX not detected” over time and vice versa. The concordance between measured MTX and the onMTX variable was 85% and the Positive Predictive Value (PPV) 100%. The association between dichotomised MTX levels and “on MTX” was highly statistically significant by logistic regression (Beta coefficient: 8.84; 95% confidence interval (0.70; 1.10); p-value: 1.3E-16).

Therefore, we used MTX levels (detectable or undetectable) to impute the missing variable onMTX (MTX status) in our dataset: where the levels were detectable for at least one time point, we decided that this patient was on MTX, when undetectable for all time points, we assumed they were not taking the drug. Consequently, the use of serum MTX levels resulted in the change from “missing” to “taking the drug” for 16 patients, and from “missing” to “not taking the drug” for 61 patients. Measured MTX levels were also used to relabel MTX use of non-adherent patients (replacing onMTX=1 by onMTX=0), where non-adherence is defined as undetectable levels of MTX in all measured timepoints. Of the 333 patients, 18 (5.5%) were deemed to be non-adherent to MTX.


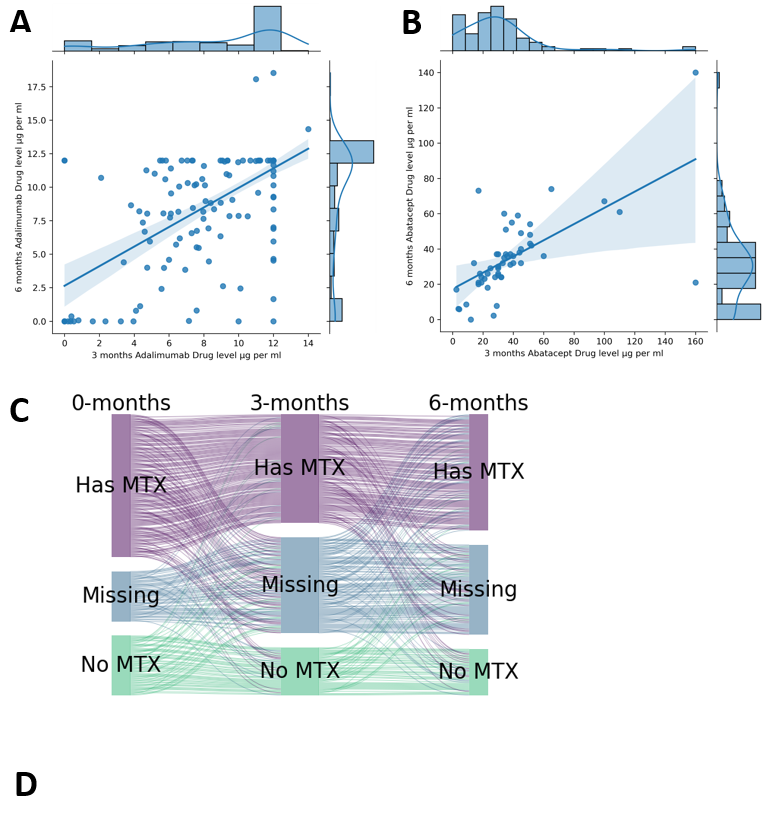


**Supplementary Figure 1: Availability and distribution of drug levels**

A) Non-trough adalimumab drug levels are well correlated between the 2 time points, but non-normally distributed, with plateau. B) The same conclusions are reached for abatacept drug levels. C) Sankey plot for the detection of serum methotrexate (MTX) at baseline (0-months), 3 months and 6 months. “Has MTX”: MTX detected in the serum. “No MTX”: MTX not detected.

|  | **At 3 months** | **At 6 months** | Correlation (3 versus 6 months) |
| --- | --- | --- | --- |
| **Adalimumab drug levels**  N available (% missing)  Concentration (µg/ml), median (IQR) | 197 (5%)  10 (5.82-12) | 184 (11%)  12 (7-12) | Spearman’s correlation:  0.65, p=1.4x10^-22^ |
| **Abatacept drug levels**  N available (% missing)  Concentration (µg/ml), median (IQR) | 94 (31%)  28.5 (16.25-36.75) | 78 (43%)  30.0 (18.5-39.5) | Spearman’s correlation:  0.73, p=8.5x10^-10^ |

**Supplementary Table 1: Availability, median levels and correlation/association between time points for biologic drug levels.** IQR: interquartile range.

|  |  | **Timepoint** | | | |
| --- | --- | --- | --- | --- | --- |
| **MTX Detected** | **onMTX** | **Baseline** | **3-months** | **6-months** | **Ever** |
| **Yes** | **Yes** | 187 | 138 | 146 | 207 (a) |
|  | **No** | 0 | 0 | 0 | 0 (c) |
|  | **Missing** | 6 | 9 | 11 | 14 |
| **No** | **Yes** | 17 | 23 | 24 | 39 (d) |
|  | **No** | 9 | 8 | 6 | 10 (b) |
|  | **Missing** | 55 | 34 | 33 | 60 |
| **Missing** | **Yes** | 17 | 60 | 51 | 102 |
|  | **No** | 44 | 45 | 47 | 49 |
|  | **Missing** | 7 | 25 | 24 | 36 |

**Supplementary Table 2: Availability of methotrexate (MTX) drug levels and concordance between detected serum levels and clinical variable**

Patient numbers at each time point where MTX was detected (Yes) or not (No), and concordance with the variable “onMTX” from clinical records (Yes: patient was prescribed MTX; no there were not). “Ever” is the total number of patients who fulfilled the criteria (f.e. MTX detected and prescribed, for the first row) at least once at any point in time. Concordance was calculated as (a+b)/(a+b+c+d)=85%. Positive Predictive Value (PPV) of measured MTX to predict onMTX: a/(a+c).

| **Covariate name** | **Beta coefficient** | **Lower CI** | **Higher CI** | **p-value** |
| --- | --- | --- | --- | --- |
| Sex | -0.27 | -0.89 | 0.34 | 0.38 |
| Age | -0.01 | -0.03 | 0.01 | 0.28 |
| Disease duration | 0.01 | -0.02 | 0.03 | 0.54 |
| Biologic-naïve | 1.45 | 0.88 | 2.01 | **5.60 x 10 ^-7^** |
| Baseline DAS28 | -0.41 | -0.68 | -0.14 | **2.8 x 10 ^-3^** |
| Comorbidity | -0.62 | -1.24 | 0.005 | 0.05 |
| MTX use | 0.62 | 0.04 | 1.21 | **0.04** |
| BMI | -0.07 | -0.12 | -0.02 | **7.5 x 10 ^-3^** |

**Supplementary Table 3: Univariate association testing of covariates with DAS28 remission**

Logistic regression was used. CI: 95% confidence interval. Beta: beta coefficient from the regression equation.

| **Covariate name** | **Beta coefficient** | **Lower CI** | **Higher CI** | **p-value** |
| --- | --- | --- | --- | --- |
| Sex | 0.08 | -0.33 | 0.48 | 0.71 |
| Age | -0.01 | -0.02 | 0.01 | 0.27 |
| Disease duration | 0.00 | -0.01 | 0.02 | 0.67 |
| Biologic-naïve | 1.07 | 0.76 | 1.39 | **8.32 x 10 ^-11^** |
| Baseline DAS28 | 0.60 | 0.43 | 0.76 | **1.04 x 10 ^-11^** |
| Comorbidity | -0.03 | -0.42 | 0.36 | 0.90 |
| MTX use | 0.49 | 0.12 | 0.85 | **8.7 x 10 ^-3^** |
| BMI | -0.03 | -0.06 | 0.00 | **0.03** |

**Supplementary Table 4: Univariate association testing of covariates with ΔDAS28**

Linear regression was used. CI: 95% confidence interval. Beta: beta coefficient from the regression equation.

| **Covariate name** | **Beta coefficient** | **Lower CI** | **Higher CI** | **p-value** |
| --- | --- | --- | --- | --- |
| Sex | -0.02 | -0.53 | 0.49 | 0.94 |
| Age | -0.01 | -0.03 | 0.01 | 0.26 |
| Disease duration | 0.00 | -0.02 | 0.02 | 0.74 |
| Biologic-naïve | 1.41 | 0.96 | 1.85 | **5.27 x 10 ^-10^** |
| Baseline DAS28 | 0.00 | -0.23 | 0.23 | 1.00 |
| Comorbidity | -0.36 | -0.83 | 0.11 | 0.14 |
| MTX use | 0.69 | 0.24 | 1.15 | **2.86 x 10 ^-3^** |
| BMI | -0.05 | -0.09 | -0.02 | **4.65 x 10 ^-3^** |

**Supplementary Table 5: Univariate association testing of covariates with EULAR response**

Ordinal regression was used. CI: 95% confidence interval. Beta: beta coefficient from the regression equation.


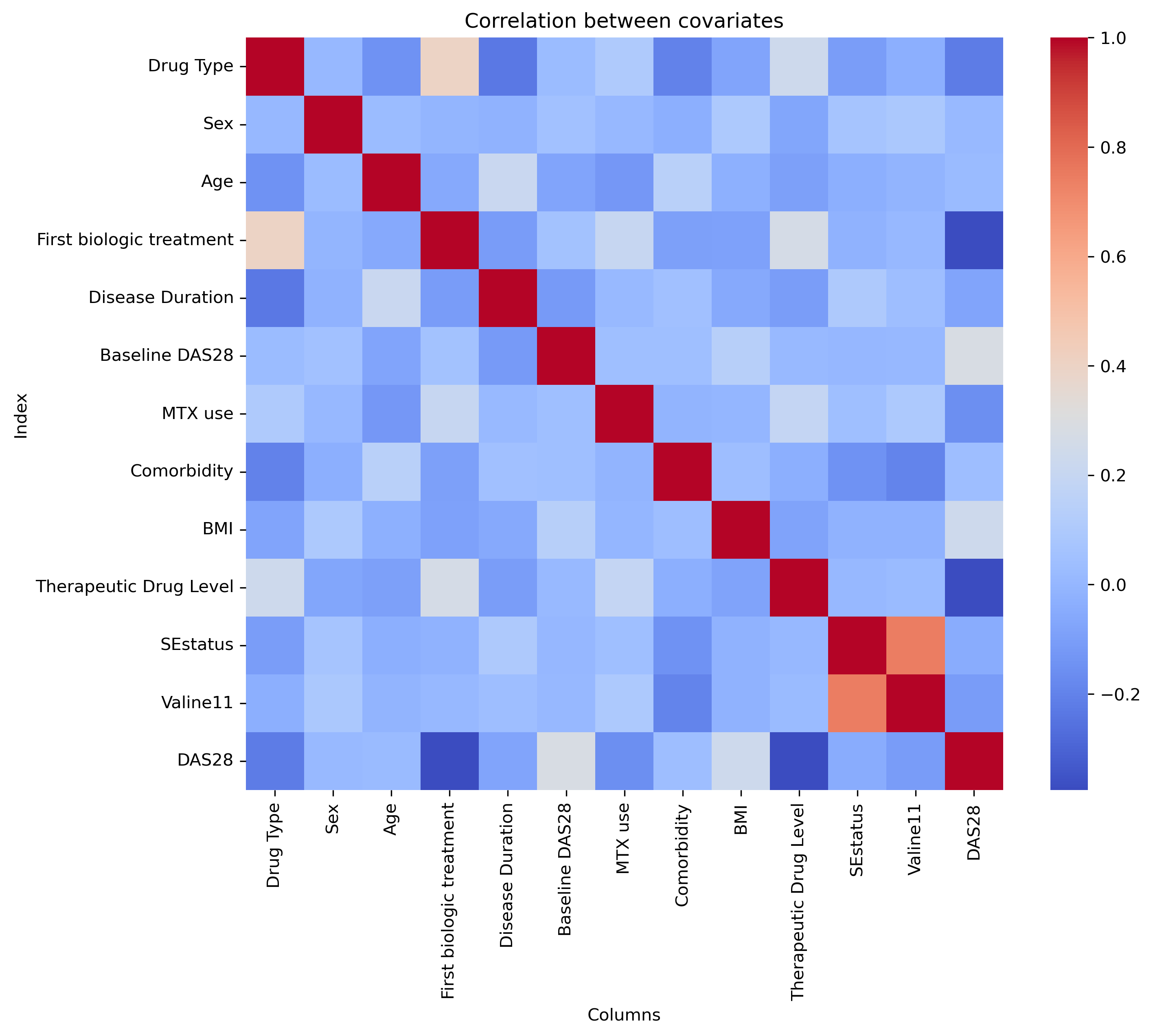


**Supplementary Figure 2: Correlation matrix heatmap**

Covariates show a low level of correlation (Pairwise Spearman correlation coefficients).

|  |  | **Remission** | | | **ΔDAS28** | | | **EULAR response** | | |
| --- | --- | --- | --- | --- | --- | --- | --- | --- | --- | --- |
|  |  | **Coef. (95% CI interval)** | **p-value** | **FMI** | **Coef. (95% CI interval)** | **p-value** | **FMI** | **Coef. (95% CI interval)** | **p-value** | **FMI** |
| **SE** | N copies of SE | 0.06 (-0.88;1.0) | 0.90 | 0.008 | 0.25 (-0.16;0.66) | 0.24 | 0.007 | 0.18 (-0.45;0.8) | 0.58 | 0.009 |
|  | Drug type | -0.78 (-3.04;1.49) | 0.50 | 0.006 | 0.42 (-0.41;1.24) | 0.32 | 0.007 | 0.05 (-1.26;1.35) | 0.94 | 0.006 |
|  | SE * Drug type | 0.82 (-0.8;2.44) | 0.32 | 0.012 | -0.1 (-0.77;0.57) | 0.78 | 0.011 | 0.07 (-0.99;1.13) | 0.90 | 0.009 |
|  | BMI | 0.01 (-0.09;0.1) | 0.90 | 0.206 | -0.02 (-0.06;0.02) | 0.27 | 0.142 | -0.04 (-0.1;0.02) | 0.16 | 0.179 |
|  | MTX use | 0.65 (-0.72;2.01) | 0.35 | 0.185 | 0.61 (0.1;1.13) | **0.02** | 0.148 | 0.89 (0.11;1.68) | **0.03** | 0.126 |
|  | Baseline DAS28 | -0.41 (-0.93;0.1) | 0.12 | 0.016 | 0.64 (0.41;0.86) | **2.8 x 10^-8^** | 0.014 | N/A | N/A | N/A |
|  | Sex | 0.04 (-1.23;1.31) | 0.95 | 0.009 | 0.25 (-0.3;0.79) | 0.38 | 0.012 | 0.21 (-0.64;1.05) | 0.63 | 0.013 |
|  | Age | -0.0 (-0.05;0.04) | 0.85 | 0.006 | 0.0 (-0.02;0.02) | 0.81 | 0.015 | 0.0 (-0.03;0.03) | 0.94 | 0.012 |
|  | Disease Duration | 0.02 (-0.04;0.07) | 0.55 | 0.015 | 0.01 (-0.01;0.03) | 0.28 | 0.021 | 0.01 (-0.03;0.04) | 0.67 | 0.031 |
|  | Therapeutic Drug Level | 1.08 (-0.06;2.21) | 0.06 | 0.004 | 0.72 (0.27;1.17) | **1.7 x 10^-3^** | 0.01 | 1.27 (0.56;1.99) | **4.9 x 10^-4^** | 0.009 |
|  | Comorbidity | 0.04 (-1.19;1.27) | 0.95 | 0.007 | 0.21 (-0.31;0.73) | 0.43 | 0.012 | 0.11 (-0.66;0.89) | 0.77 | 0.011 |
| **Val11** | N copies of Val11 | -0.08 (-1.03;0.87) | 0.87 | 0.01 | 0.19 (-0.23;0.61) | 0.37 | 0.013 | 0.59 (-0.06;1.24) | 0.08 | 0.019 |
|  | Drug type | -0.3 (-2.04;1.43) | 0.73 | 0.004 | 0.25 (-0.44;0.94) | 0.48 | 0.009 | 0.24 (-0.87;1.35) | 0.67 | 0.009 |
|  | Val11*Drug type | 0.52 (-0.97;2.02) | 0.49 | 0.003 | 0.08 (-0.59;0.75) | 0.81 | 0.007 | -0.1 (-1.13;0.93) | 0.84 | 0.01 |
|  | BMI | 0.0 (-0.09;0.09) | 0.99 | 0.204 | -0.02 (-0.06;0.01) | 0.23 | 0.149 | -0.05 (-0.11;0.01) | 0.12 | 0.248 |
|  | MTX use | 0.62 (-0.74;1.99) | 0.37 | 0.19 | 0.56 (0.03;1.08) | **0.04** | 0.148 | 0.76 (-0.06;1.58) | 0.07 | 0.168 |
|  | Baseline DAS28 | -0.47 (-0.96;0.03) | 0.07 | 0.015 | 0.63 (0.41;0.86) | **3.05 x 10^-8^** | 0.012 | N/A | N/A | N/A |
|  | Sex | 0.11 (-1.15;1.37) | 0.87 | 0.009 | 0.22 (-0.32;0.77) | 0.43 | 0.011 | 0.09 (-0.77;0.95) | 0.84 | 0.009 |
|  | Age | -0.0 (-0.05;0.05) | 0.93 | 0.005 | 0.0 (-0.02;0.02) | 1.00 | 0.016 | -0.0 (-0.04;0.03) | 0.76 | 0.015 |
|  | Disease Duration | 0.02 (-0.04;0.07) | 0.54 | 0.016 | 0.01 (-0.01;0.04) | 0.20 | 0.021 | 0.01 (-0.02;0.04) | 0.56 | 0.037 |
|  | Therapeutic Drug Level | 1.1 (-0.03;2.23) | 0.06 | 0.004 | 0.71 (0.26;1.17) | **2 x 10^-3^** | 0.009 | 1.28 (0.56;2.0) | **5 x 10^-4^** | 0.008 |
|  | Comorbidity | -0.01 (-1.27;1.24) | 0.98 | 0.008 | 0.23 (-0.3;0.75) | 0.40 | 0.013 | 0.27 (-0.52;1.06) | 0.50 | 0.01 |

**Supplementary Table 6: Association testing of genetic factors and drug type with response to treatment, restricted to patients starting a second line (or higher order) biologic treatment (total number of non-biologic-naïve patients: 162).** FMI stands for fraction of missing information. This analysis complements Table 2, see legend for further information. P-values below the nominal significance level of 0.05 (no correction for multiple testing) are highlighted in bold. No difference in the efficacy of adalimumab or abatacept is observed. The interaction term (N copies of SE *Drug type or N copies of Val11*Drug type) is not significant, therefore the effect of genetic factors on response to treatment is similar for both drugs. N/A: models for EULAR response were not adjusted for Baseline DAS28, as this variable is included in the definition of EULAR response (see methods). N: number.

|  |  | **Remission** | | | **ΔDAS28** | | | **EULAR response** | | |
| --- | --- | --- | --- | --- | --- | --- | --- | --- | --- | --- |
|  |  | **Coef. (95% CI interval)** | **p-value** | **FMI** | **Coef. (95% CI interval)** | **p-value** | **FMI** | **Coef. (95% CI interval)** | **p-value** | **FMI** |
| **SE** | N copies of SE | 1.64 (0.14;3.14) | **0.03** | 0.01 | 0.53 (-0.09;1.15) | 0.10 | 0.016 | 0.74 (-0.29;1.77) | 0.16 | 0.007 |
|  | Drug type | 2.17 (-0.27;4.61) | 0.08 | 0.007 | 0.35 (-0.61;1.31) | 0.47 | 0.014 | 0.86 (-0.62;2.33) | 0.25 | 0.007 |
|  | SE * Drug type | -1.85 (-3.47;-0.24) | **0.02** | 0.009 | -0.49 (-1.18;0.2) | 0.16 | 0.013 | -0.81 (-1.94;0.33) | 0.16 | 0.005 |
|  | BMI | -0.08 (-0.15;-0.0) | **0.04** | 0.143 | -0.04 (-0.07;-0.01) | **0.02** | 0.119 | -0.05 (-0.1;0.01) | 0.12 | 0.129 |
|  | MTX use | 0.18 (-0.83;1.19) | 0.73 | 0.112 | 0.09 (-0.43;0.62) | 0.72 | 0.125 | 0.17 (-0.76;1.1) | 0.72 | 0.119 |
|  | Baseline DAS28 | -0.47 (-0.9;-0.04) | **0.03** | 0.015 | 0.63 (0.42;0.85) | **5.75 x 10^-9^** | 0.01 | N/A | N/A | N/A |
|  | Sex | -0.18 (-1.03;0.67) | 0.68 | 0.01 | -0.02 (-0.46;0.41) | 0.91 | 0.007 | -0.02 (-0.77;0.73) | 0.95 | 0.005 |
|  | Age | -0.01 (-0.04;0.02) | 0.72 | 0.006 | -0.0 (-0.02;0.01) | 0.74 | 0.009 | -0.0 (-0.03;0.02) | 0.82 | 0.008 |
|  | Disease Duration | 0.01 (-0.03;0.04) | 0.68 | 0.023 | 0.02 (-0.0;0.04) | 0.08 | 0.015 | 0.02 (-0.02;0.05) | 0.35 | 0.014 |
|  | Therapeutic Drug Level | 1.32 (0.29;2.35) | **0.01** | 0.009 | 0.76 (0.29;1.23) | **1.5 x 10^-3^** | 0.015 | 1.13 (0.32;1.94) | **6.46 x 10^-3^** | 0.009 |
|  | Comorbidity | -0.66 (-1.59;0.26) | 0.16 | 0.004 | -0.02 (-0.46;0.43) | 0.94 | 0.005 | -0.29 (-1.06;0.48) | 0.46 | 0.003 |
| **Val11** | N copies of Val11 | 2.17 (0.42;3.92) | **0.02** | 0.006 | 0.41 (-0.22;1.04) | 0.20 | 0.013 | 1.01 (-0.15;2.17) | 0.09 | 0.004 |
|  | Drug type | 2.08 (-0.23;4.38) | 0.08 | 0.005 | 0.07 (-0.76;0.89) | 0.88 | 0.014 | 0.81 (-0.52;2.13) | 0.23 | 0.006 |
|  | Val11*Drug type | -2.1 (-3.94;-0.26) | **0.02** | 0.006 | -0.3 (-0.99;0.38) | 0.38 | 0.011 | -1.0 (-2.24;0.24) | 0.11 | 0.003 |
|  | BMI | -0.08 (-0.15;-0.0) | **0.04** | 0.138 | -0.04 (-0.07;-0.0) | **0.03** | 0.113 | -0.04 (-0.1;0.02) | 0.15 | 0.118 |
|  | MTX use | 0.38 (-0.66;1.43) | 0.47 | 0.12 | 0.09 (-0.43;0.62) | 0.72 | 0.111 | 0.18 (-0.75;1.12) | 0.70 | 0.117 |
|  | Baseline DAS28 | -0.47 (-0.9;-0.05) | **0.03** | 0.014 | 0.63 (0.42;0.84) | **6.21 x 10^-9^** | 0.01 | N/A | N/A | N/A |
|  | Sex | -0.21 (-1.07;0.64) | 0.63 | 0.01 | -0.02 (-0.46;0.41) | 0.92 | 0.008 | -0.06 (-0.81;0.7) | 0.88 | 0.005 |
|  | Age | -0.0 (-0.03;0.03) | 0.77 | 0.007 | -0.0 (-0.02;0.01) | 0.71 | 0.007 | -0.0 (-0.03;0.02) | 0.82 | 0.006 |
|  | Disease Duration | 0.01 (-0.03;0.04) | 0.77 | 0.021 | 0.02 (-0.0;0.04) | 0.07 | 0.013 | 0.02 (-0.02;0.05) | 0.34 | 0.015 |
|  | Therapeutic Drug Level | 1.16 (0.13;2.19) | **0.03** | 0.008 | 0.72 (0.25;1.18) | **2.6 x 10^-3^** | 0.012 | 1.08 (0.27;1.88) | **8.79 x 10^-3^** | 0.008 |
|  | Comorbidity | -0.5 (-1.42;0.43) | 0.29 | 0.004 | -0.01 (-0.46;0.43) | 0.96 | 0.005 | -0.27 (-1.04;0.5) | 0.50 | 0.003 |

**Supplementary Table 7: Association testing of genetic factors and drug type with response to treatment, restricted to biologic-naïve patients (total patient number: 180).** FMI stands for fraction of missing information. This analysis complements Table 2, see legend for further information. P-values below the nominal significance level of 0.05 (no correction for multiple testing) are highlighted in bold. No difference in the efficacy of adalimumab or abatacept is observed. Carriers of the SE or Val11 are more likely to enter remission than non-carriers, and this effect is statistically significantly different between the two drugs, with carriers significantly more likely to enter remission when treated with abatacept than when treated with adalimumab (the interaction term (N copies of SE *Drug type or N copies of Val11*Drug type) is significant, therefore there is a statistically significant differential effectiveness of genetic factors on remission for the two drugs in biologic-naïve patients). However, this analysis is based on very small absolute patient numbers (see breakdown in **Supplementary Figure 3**).

A)


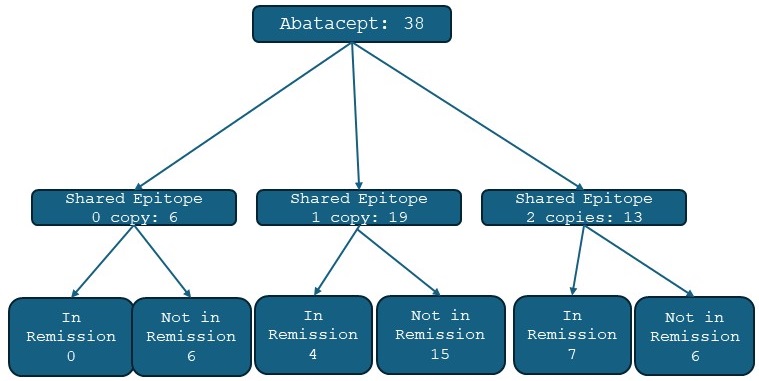


B)


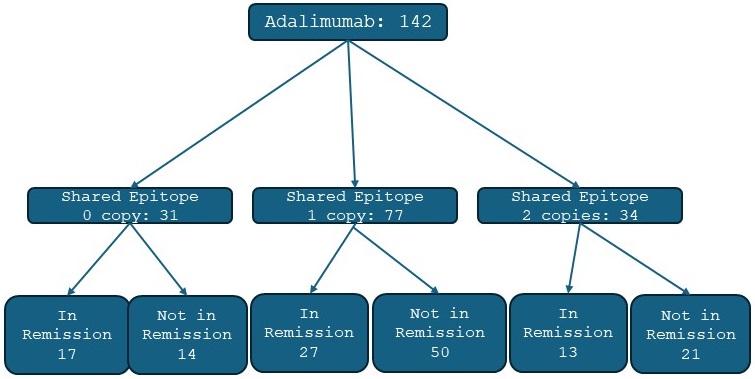


**Supplementary Figure 3: Breakdown of the 180 biologic-naïve patients analysed in Supplementary Table 7.**

38 patients received abatacept as first line biologic drug (A), while 142 received adalimumab as first line choice. Numbers in each box refer to number of patients within the parental category satisfying the condition outlined in the box and therefore included in the analysis presented in Supplementary Table 7.

|  |  | **Remission** | | | **ΔDAS28** | | | **EULAR response** | | |
| --- | --- | --- | --- | --- | --- | --- | --- | --- | --- | --- |
|  |  | **Coef. (95% CI interval)** | **p-value** | **FMI** | **Coef. (95% CI interval)** | **p-value** | **FMI** | **Coef. (95% CI interval)** | **p-value** | **FMI** |
| **SE** | N copies of SE | 0.58 (-0.17;1.32) | 0.13 | 0.006 | 0.33 (-0.0;0.66) | 0.05 | 0.007 | 0.32 (-0.2;0.84) | 0.23 | 0.006 |
|  | Drug type | 0.65 (-0.72;2.03) | 0.35 | 0.006 | 0.36 (-0.22;0.95) | 0.22 | 0.008 | 0.41 (-0.52;1.35) | 0.39 | 0.005 |
|  | SE * Drug type | -0.59 (-1.47;0.3) | 0.20 | 0.004 | -0.24 (-0.65;0.17) | 0.25 | 0.008 | -0.28 (-0.94;0.38) | 0.41 | 0.005 |
|  | BMI | -0.05 (-0.1;0.01) | 0.11 | 0.191 | -0.03 (-0.05;-0.0) | **0.02** | 0.155 | -0.05 (-0.09;-0.01) | **0.03** | 0.188 |
|  | MTX use | 0.35 (-0.43;1.14) | 0.38 | 0.168 | 0.37 (0.01;0.73) | **0.04** | 0.166 | 0.57 (-0.02;1.16) | 0.06 | 0.156 |
|  | Biologic-naïve | 1.3 (0.61;1.98) | **2 x 10^-4^** | 0.005 | 0.77 (0.45;1.09) | **2.41 x 10^-6^** | 0.009 | 1.15 (0.63;1.68) | **2.00 x 10^-5^** | 0.007 |
|  | Baseline DAS28 | -0.52 (-0.83;-0.2) | **1.2 x 10^-3^** | 0.011 | 0.61 (0.46;0.76) | **1.88 x 10^-15^** | 0.008 | N/A | N/A | N/A |
|  | Sex | -0.11 (-0.8;0.59) | 0.76 | 0.01 | 0.06 (-0.27;0.39) | 0.73 | 0.006 | 0.05 (-0.5;0.6) | 0.86 | 0.008 |
|  | Age | -0.01 (-0.03;0.02) | 0.67 | 0.006 | -0.0 (-0.01;0.01) | 0.82 | 0.006 | -0.0 (-0.02;0.02) | 0.83 | 0.006 |
|  | Disease Duration | 0.01 (-0.02;0.04) | 0.46 | 0.019 | 0.01 (0.0;0.03) | **0.03** | 0.019 | 0.01 (-0.01;0.04) | 0.26 | 0.021 |
|  | Therapeutic Drug Level | 1.11 (0.38;1.84) | **2.9 x 10^-3^** | 0.007 | 0.7 (0.38;1.02) | **1.64 x 10^-5^** | 0.009 | 1.14 (0.62;1.67) | **2.00 x 10^-5^** | 0.01 |
|  | Calendar Date | -0.0 (-0.0;0.0) | 0.83 | 0.005 | 0.0 (-0.0;0.0) | 0.96 | 0.01 | 0.0 (-0.0;0.0) | 0.67 | 0.007 |
|  | Comorbidity | -0.37 (-1.09;0.35) | 0.32 | 0.004 | 0.08 (-0.26;0.41) | 0.65 | 0.007 | -0.12 (-0.66;0.41) | 0.65 | 0.006 |
| **Val11** | N copies of Val11 | 0.62 (-0.1;1.35) | 0.09 | 0.002 | 0.29 (-0.04;0.62) | 0.08 | 0.007 | 0.66 (0.12;1.19) | **0.02** | 0.006 |
|  | Drug type | 0.43 (-0.77;1.64) | 0.48 | 0.005 | 0.2 (-0.28;0.68) | 0.41 | 0.009 | 0.59 (-0.25;1.44) | 0.17 | 0.006 |
|  | Val11*Drug type | -0.47 (-1.34;0.4) | 0.29 | 0.002 | -0.13 (-0.53;0.28) | 0.54 | 0.006 | -0.52 (-1.19;0.14) | 0.12 | 0.006 |
|  | BMI | -0.05 (-0.1;0.01) | 0.10 | 0.185 | -0.03 (-0.05;-0.0) | **0.02** | 0.153 | -0.05 (-0.09;-0.01) | **0.03** | 0.166 |
|  | MTX use | 0.34 (-0.45;1.13) | 0.39 | 0.171 | 0.33 (-0.03;0.69) | 0.07 | 0.166 | 0.54 (-0.05;1.12) | 0.07 | 0.154 |
|  | Biologic-naïve | 1.3 (0.61;1.98) | **2 x 10^-4^** | 0.005 | 0.77 (0.46;1.09) | **1.47 x 10^-6^** | 0.009 | 1.19 (0.66;1.71) | **1.00 x 10^-5^** | 0.006 |
|  | Baseline DAS28 | -0.53 (-0.84;-0.21) | **1 x 10^-3^** | 0.01 | 0.6 (0.45;0.75) | **1.48x 10^-15^** | 0.007 | N/A | N/A | N/A |
|  | Sex | -0.12 (-0.81;0.57) | 0.73 | 0.01 | 0.05 (-0.28;0.38) | 0.77 | 0.005 | -0.01 (-0.56;0.54) | 0.97 | 0.008 |
|  | Age | -0.01 (-0.03;0.02) | 0.62 | 0.006 | -0.0 (-0.01;0.01) | 0.68 | 0.006 | -0.0 (-0.02;0.02) | 0.67 | 0.007 |
|  | Disease Duration | 0.01 (-0.02;0.04) | 0.45 | 0.019 | 0.02 (0.0;0.03) | **0.02** | 0.019 | 0.01 (-0.01;0.04) | 0.22 | 0.017 |
|  | Therapeutic Drug Level | 1.09 (0.36;1.82) | **3.4 x 10^-3^** | 0.007 | 0.69 (0.37;1.0) | **2.07 x 10^-5^** | 0.009 | 1.14 (0.62;1.66) | **2.00 x 10^-5^** | 0.006 |
|  | Calendar Date | -0.0 (-0.0;0.0) | 0.90 | 0.004 | 0.09 (-0.24;0.43) | 0.58 | 0.006 | 0.0 (-0.0;0.0) | 0.49 | 0.008 |
|  | Comorbidity | -0.31 (-1.04;0.41) | 0.40 | 0.004 | 0.29 (-0.04;0.62) | 0.08 | 0.007 | -0.06 (-0.6;0.48) | 0.83 | 0.005 |

**Supplementary Table 8: Association testing of genetic factors and drug type with response to treatment adjusted for calendar date.**

FMI stands for fraction of missing information. This analysis complements Table 2, see legend for further information. P-values below the nominal significance level of 0.05 (no correction for multiple testing) are highlighted in bold. Calendar date is not significantly associated with any measure of response to treatment. Therefore, results presented in this table align with those presented in Table 2: the multivariate model shows an independent association between the carriage of Val11 and EULAR response, but no evidence of independent association of drug type or other genetic markers with other measures of response to treatment.

|  |  | **Remission** | | | **ΔDAS28** | | | **EULAR response** | | |
| --- | --- | --- | --- | --- | --- | --- | --- | --- | --- | --- |
|  |  | **Coef. (95% CI interval)** | **p-value** | **FMI** | **Coef. (95% CI interval)** | **p-value** | **FMI** | **Coef. (95% CI interval)** | **p-value** | **FMI** |
| **SE** | N copies of SE | -1.03 (-3.15;1.1) | 0.34 | 0.005 | -0.96 (-1.99;0.08) | 0.07 | 0.009 | -1.56 (-3.45;0.34) | 0.11 | 0.012 |
|  | Drug type | -1.53 (-5.34;2.28) | 0.43 | 0.007 | -0.82 (-2.6;0.96) | 0.37 | 0.01 | -2.92 (-6.25;0.41) | 0.09 | 0.01 |
|  | SE*Drug type | 0.85 (-1.36;3.06) | 0.45 | 0.006 | 0.96 (-0.12;2.03) | 0.08 | 0.009 | 1.83 (-0.19;3.84) | 0.08 | 0.011 |
|  | BMI | -0.01 (-0.1;0.08) | 0.84 | 0.104 | -0.02 (-0.06;0.03) | 0.48 | 0.095 | -0.04 (-0.12;0.04) | 0.37 | 0.089 |
|  | MTX use | 0.57 (-0.94;2.08) | 0.46 | 0.142 | 0.39 (-0.27;1.04) | 0.25 | 0.057 | 0.87 (-0.3;2.04) | 0.14 | 0.049 |
|  | Biologic-naïve | 1.25 (-0.14;2.65) | 0.08 | 0.004 | 0.33 (-0.28;0.95) | 0.29 | 0.004 | 1.31 (0.22;2.41) | **0.02** | 0.005 |
|  | Baseline DAS28 | -0.58 (-1.1;-0.05) | **0.03** | 0.003 | 0.71 (0.48;0.94) | **1.55 x 10 ^-9^** | 0.004 | N/A | N/A | N/A |
|  | Sex | 0.67 (-0.41;1.74) | 0.22 | 0.004 | 0.19 (-0.3;0.69) | 0.45 | 0.006 | 0.11 (-0.83;1.06) | 0.81 | 0.009 |
|  | Age | 0.0 (-0.04;0.04) | 0.96 | 0.009 | 0.01 (-0.01;0.03) | 0.38 | 0.003 | 0.02 (-0.02;0.05) | 0.40 | 0.003 |
|  | Disease Duration | 0.02 (-0.03;0.07) | 0.36 | 0.013 | 0.03 (0.0;0.05) | **0.03** | 0.026 | 0.03 (-0.02;0.08) | 0.26 | 0.041 |
|  | Drug Therapeutic Level | 1.22 (-0.13;2.56) | 0.08 | 0.003 | 1.05 (0.5;1.61) | **2 x 10 ^-4^** | 0.005 | 1.63 (0.57;2.7) | **2.69 x 10 ^-3^** | 0.003 |
|  | Comorbidity | -0.94 (-2.58;0.7) | 0.26 | 0.001 | -0.46 (-1.2;0.29) | 0.23 | 0.004 | -1.2 (-2.64;0.24) | 0.10 | 0.004 |
| **Val11** | N copies of Val11 | -0.0 (-2.27;2.26) | 1.00 | 0.002 | -0.46 (-1.39;0.48) | 0.34 | 0.003 | 0.06 (-1.58;1.71) | 0.94 | 0.004 |
|  | Drug type | -0.15 (-3.8;3.5) | 0.94 | 0.004 | -0.14 (-1.69;1.4) | 0.86 | 0.005 | -0.43 (-3.19;2.33) | 0.76 | 0.004 |
|  | Val11*Drug type | 0.12 (-2.23;2.46) | 0.92 | 0.002 | 0.62 (-0.37;1.61) | 0.22 | 0.003 | 0.36 (-1.4;2.12) | 0.69 | 0.004 |
|  | BMI | -0.01 (-0.1;0.08) | 0.89 | 0.106 | -0.01 (-0.06;0.03) | 0.59 | 0.094 | -0.03 (-0.11;0.06) | 0.50 | 0.118 |
|  | MTX use | 0.6 (-0.9;2.11) | 0.43 | 0.158 | 0.39 (-0.27;1.06) | 0.27 | 0.084 | 0.76 (-0.43;1.96) | 0.21 | 0.084 |
|  | Biologic-naïve | 1.21 (-0.18;2.61) | 0.09 | 0.004 | 0.32 (-0.3;0.95) | 0.31 | 0.004 | 1.16 (0.06;2.26) | **0.04** | 0.005 |
|  | Baseline DAS28 | -0.58 (-1.1;-0.06) | **0.03** | 0.003 | 0.69 (0.46;0.93) | **5.92 x 10^-9^** | 0.004 | N/A | N/A | N/A |
|  | Sex | 0.7 (-0.37;1.77) | 0.20 | 0.004 | 0.2 (-0.3;0.7) | 0.44 | 0.006 | 0.05 (-0.88;0.99) | 0.91 | 0.004 |
|  | Age | 0.01 (-0.04;0.05) | 0.80 | 0.008 | 0.01 (-0.01;0.03) | 0.24 | 0.004 | 0.02 (-0.02;0.06) | 0.41 | 0.005 |
|  | Disease Duration | 0.02 (-0.03;0.07) | 0.36 | 0.013 | 0.03 (0.0;0.05) | **0.04** | 0.026 | 0.02 (-0.02;0.07) | 0.31 | 0.034 |
|  | Drug Therapeutic Level | 1.18 (-0.16;2.52) | 0.08 | 0.003 | 1.02 (0.46;1.58) | **4 x 10^-4^** | 0.004 | 1.6 (0.55;2.66) | **2.98 x 10 ^-3^** | 0.003 |
|  | Comorbidity | -0.63 (-2.27;1.01) | 0.45 | 0.002 | -0.31 (-1.08;0.46) | 0.43 | 0.005 | -0.7 (-2.2;0.79) | 0.36 | 0.005 |

**Supplementary Table 9: Association testing of genetic factors and drug type with response to treatment in ACPA-positive patients (n=125).**

FMI stands for fraction of missing information. This analysis complements Table 2, see legend for further information. P-values below the nominal significance level of 0.05 (no correction for multiple testing) are highlighted in bold. Multivariate model shows no evidence of independent association of genetic markers or drug type with response to treatment.
